# Thermodynamics of Self-Assembly of Dicarboxylate Ions with Binuclear Lanthanide Complexes

**DOI:** 10.1002/open.201500060

**Published:** 2015-06-25

**Authors:** Thomas Just Sørensen, Leila R Hill, Stephen Faulkner

**Affiliations:** a Nanoscience Centre and Department of Chemistry, University of CopenhagenUniversitetsparken 5, 2100, København Ø, Denmark; b Chemistry Research Laboratory, Oxford University12 Mansfield Road, Oxford, OX1 3TA, UK

**Keywords:** lanthanides, luminescence, self-assembly, supramolecular chemistry

## Abstract

Self-assembly of a range of carboxylic acids (benzoic acid, dinicotinic acid, nicotinic acid, and isophthalic acid) with the europium complex of 5-nitro-α,α′-bis(DO3Ayl)-*m*-xylene (where DO3A is 1,4,7,10-tetraazacyclododecane-1,4,7-triacetic acid) has been explored to establish the thermodynamics of binding in a range of solvent systems and in a range of aqueous buffer solutions. In this system, profound effects are observed as a consequence of competition by the hydroxide ion, which outcompetes even dinicotinate at high pH. In the case of isophthalate, which binds most strongly, and dinicotinate, both enthalpic and entropic contributions to binding have been identified. The europium complex with 5-nitro-α,α′-bis(DO3Ayl)-*m*-xylene is found to have a solution structure significantly different from the related europium complex of 5-amino-α,α′-bis(DO3Ayl)-*m*-xylene. It is found that phosphate binds strongly to the europium complex of the nitro derivate but not to the europium complex of amino derivative. Lactate, citrate, and pyruvate also bind strongly to 5-nitro-α,α′-bis(Eu⋅DO3Ayl)-*m*-xylene, and it is concluded that the solution structure of this binuclear lanthanide complex is significantly different from that of the amino-substituted complex.

## Introduction

The use of lanthanide complexes to define the concentration of a range of analytes is well established, both in terms of supramolecular coordination chemistry and bioassays. In many of these applications, direct coordination of anions to the lanthanide can be exploited to give concentration-dependent variations in the photophysical properties at the lanthanide centre.^1^–^5^ Due to the nature of the 4*f* orbitals, the interactions between the analytes and trivalent lanthanide ions exploited in these applications are predominately ionic in nature.^6^

By using time-gated techniques, the long-lived luminescence from lanthanide ions can be exploited to give low detection limits in microscopy and assays when nearby chromophores are used to sensitise the formation of the lanthanide excited state.^7^–^15^ In such systems, there are many ways in which the lanthanide luminescence can be perturbed by an analyte. In the majority of systems, sensitised luminescence occurs via formation of an excited singlet state on the chromophore, followed by intersystem crossing to the triplet, and subsequent energy transfer to the lanthanide emissive state; all of these intermediate states can be perturbed by collisional quenching or association with a guest to form a ternary complex.^16^–^18^ For instance, lanthanide complexes with heptadentate ligands derived from 1,4,7,10-tetraazacyclododecane-1,4,7-triacetic acid (DO3A) form complexes with a wide range of substrates.^19^–^21^ Such interactions have been used to exploit chelating interactions with a range of bidentate anions to achieve changes to lanthanide luminescence by displacing solvent.^22^–^25^ Related approaches have been used to screen sensitising chromophores and in the development of displacement assays.^21^,^26^

In our case, we have focused on the interaction between stable binuclear lanthanide complexes and a range of bidentate anions. Initially, we established that lanthanide complexes of α,α′-(DO3Ayl)-*m*-xylene are selective for isophthalate over terephthalate and phthalate.^27^ Subsequently, we have explored the binding of water-soluble guests in such systems,^28^ and established that such complexes can be recruited to isophthalate-functionalised surfaces.^29^ The isophthalate motif can also be used to direct the assembly of more complicated multimetallic *f,f*’ and *d*,*f* complexes.^27^,^30^ We recently observed that changing the remote substituent on the *m*-xylyl linking group can be used to control the binding affinity of the complex for an isophthalate guest, and that binding can be precluded or enhanced depending on the nature of the remote substituent.^31^ Affinity constants (*K*) between 0 and 10^7^ m^−1^ were observed, and the considerable changes in this system were ascribed to preorganisation of the binuclear complex and removal of “inactive” conformers from the conformational space available within the structure.^31^ Exclusion of the “active” conformer in such circumstances effectively precludes binding.^32^

**Scheme 1:**
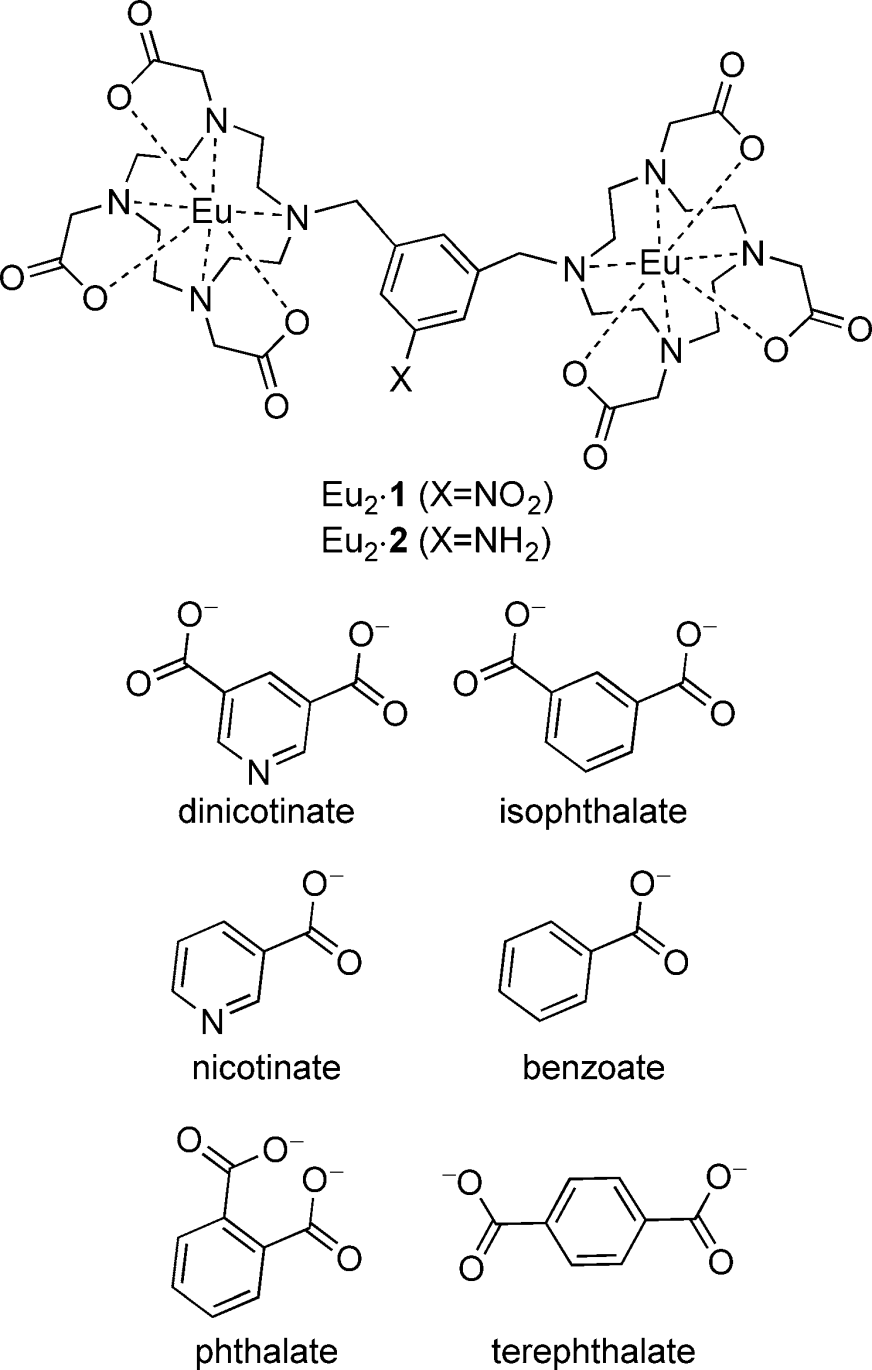
Molecules and lanthanide complexes studied: 5-nitro-α,α′-bis(Eu⋅ DO3Ayl)-*m*-xylene (Eu_2_⋅1) and 5-amino-α,α′-bis(Eu⋅DO3Ayl)-*m*-xylene (Eu_2_⋅2).

These observations, together with the fact that solvent plays a dominant role in determining the value of the affinity constant,^33^ made us consider the thermodynamics of binding in detail. Here, we describe the results of a study in which we look at the role of solvent and buffer, and establish enthalpic and entropic contributions to the thermodynamics of anion binding. We show that the enthalpic and entropic contributions change sign when going from methanol to water, and document that guest binding at a lanthanide centre can occur with no displacement of quenching O−H oscillators. Our investigations into the binding of a series of binuclear complexes with the isomers of phthalic acid in methanolic solution (Scheme 1) show that the small differences between the complexes Eu_2_⋅**1** and Eu_2_⋅**2** have dramatic effects in host–guest equilibria; substituting an amino group for a nitro group results in reversal of observed selectivities. Furthermore, uncompetitive media for Eu_2_⋅**2** can be competitive with guests for Eu_2_⋅**1** to the degree that no host–guest interaction is observed in biologically relevant media.

## Results and Discussion

5-nitro-α,α′-(DO3Ayl)-*m*-xylene (H_6_⋅**1**) and its binuclear europium complex (Eu_2_⋅**1**) were prepared as previously described.^34^ Although this ligand was among the first *m*-xylyl-bridged bismacrocycles to be prepared, we have previously only carried out very limited studies upon the binding of anionic guest species beyond establishing the affinity for dinicotinate in tris(hydroxymethyl)aminomethane (TRIS) buffer.^28^ In this study, we report a much broader range of data and establish the affinity for four different anions, and define the binding in methanolic and aqueous media at a range of temperatures.

### Studying self-assembly

Titrations of Eu_2_⋅**1** were carried out with the anions shown in Scheme 1 using a previously described procedure.^33^ Briefly, a 10^−5^ m solution of the lanthanide complex in the solvent system under study was used to prepare a 10^−3^ m solution of the carboxylate species, containing 10^−5^ m of the lanthanide complex. In doing so, the concentration of the lanthanide complex remained constant during the titration. A 2 mL aliquot of the stock solution was placed in a cuvette, and the luminescence spectrum recorded.

**Figure 1:**
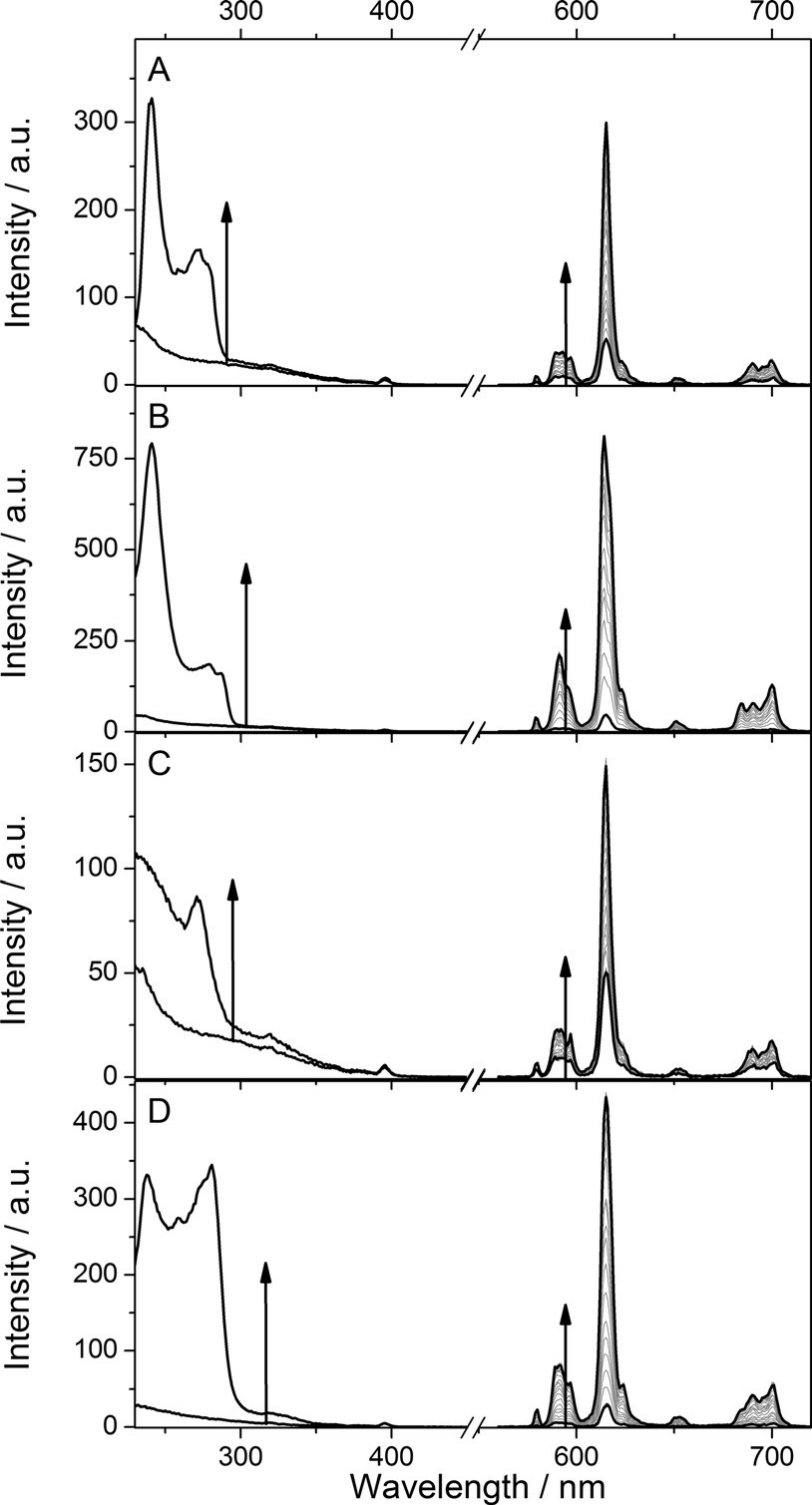
Titrations of 5-nitro-α,α′-bis(Eu⋅DO3Ayl)-*m*-xylene (Eu_2_⋅1; 0.011 mm) with A) benzoate (1.33 mm), B) isophthalate (2.15 mm), C) nicotinate (1.59 mm), and D) dinicotinate (1.01 mm) at 25 °C in methanol with 1 mm LiOH⋅H_2_O followed using time-gated emission spectroscopy with excitation at 240 nm. The end points are shown in bold black lines. The end-point excitation spectra recorded by following 620 nm emission are shown in bold.

**Figure 2:**
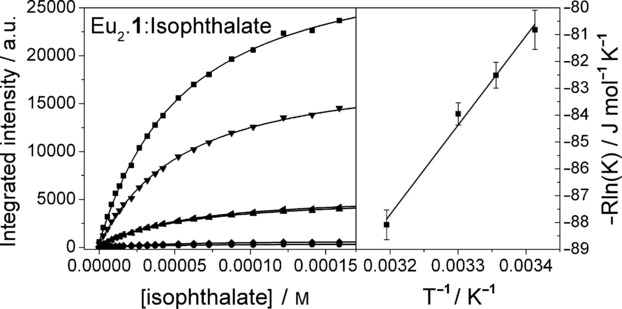
Left: Titration isotherms from a titration of 5-nitro-α,α′-bis(Eu⋅ DO3Ayl)-*m*-xylene (Eu_2_⋅1; 0.011 mm) with isophthalate (*R*^2^=0.987; 2.15 mm) at 25 °C in methanol with 1 mm LiOH⋅H_2_O determined by recording emission spectra following excitation at 240 nm. The isotherms show integrated emission bands [*I*_total_ (▪), Δ*J* = 0 (•), 1 (▴), 2 (▾), 3 (♦), 4 (◂)] and the best fit to the data (—). Right: A van′t Hoff plot of data from titrations performed at several temperatures (20, 25, 30, and 40 °C).

**Figure 3:**
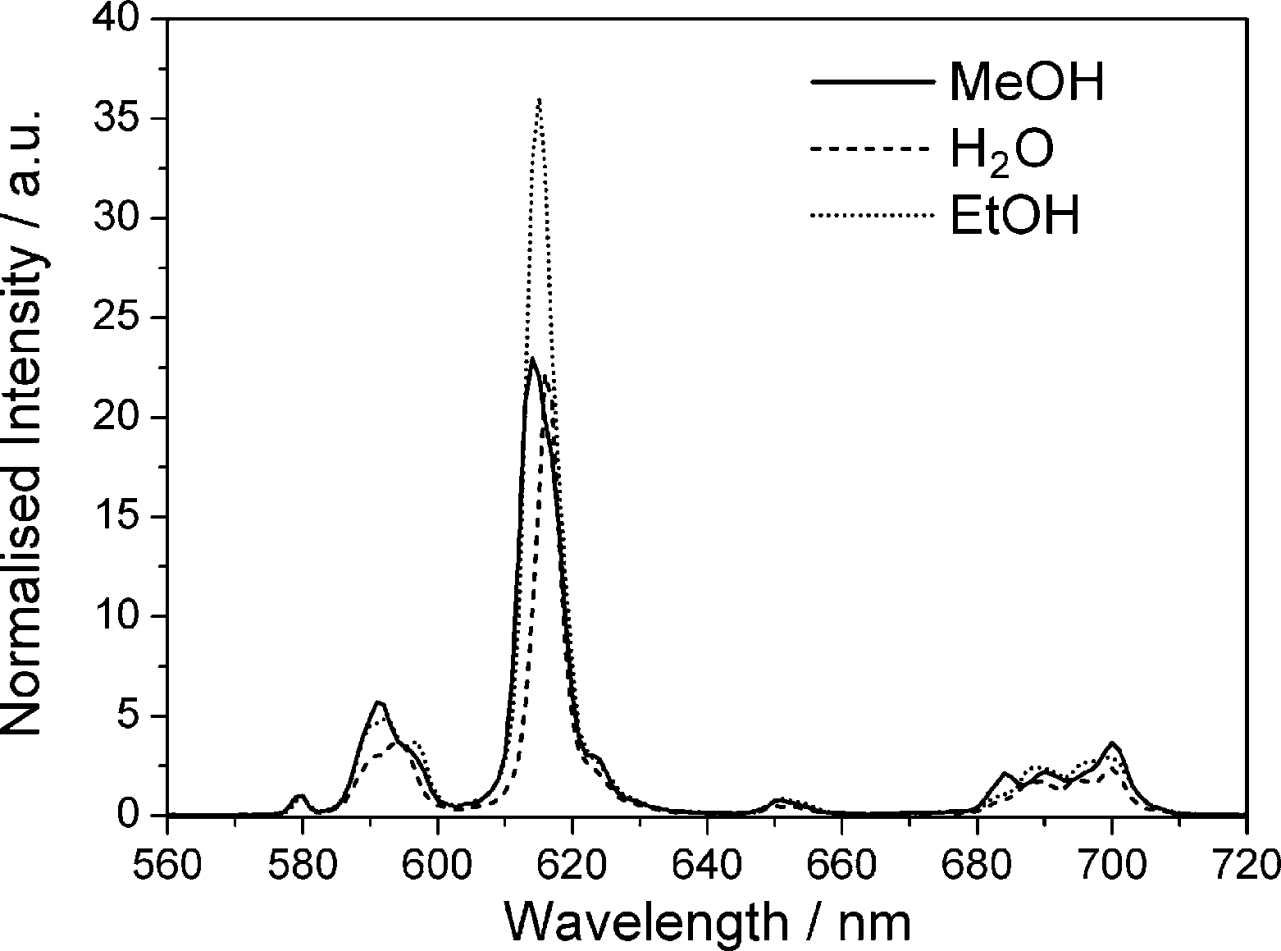
Time-gated emission spectra measured following 240 nm excitation of 5-nitro-α,α′-bis(Eu⋅DO3Ayl)-*m*-xylene (Eu_2_⋅1).

An aliquot, ranging from 2 μL to 100 μL, of the titrant was added, and the luminescence spectrum recorded again. The titration was continued until the binding isotherm had reached a plateau. The results of these studies are summarised in Figures 1, 2 and 3, and Tables 1 and 2, and will be discussed in greater detail below.

Figure 1 shows the changes in excitation and emission spectra during the course of a titration of Eu_2_⋅**1** with the anions investigated in this study: benzoate, isophthalate, nicotinate, and dinicotinate. Two different aromatic bridging rings, derived from pyridine and benzene with one and two carboxylate groups, were investigated. Cursory inspection of the data shows that no significant changes in the band shape is apparent in the spectrum; data from all titrations performed in this study can be found in the Supporting Information.

### Determining binding constants

First, the four guests were compared in methanol, a solvent in which all guest molecules are fully soluble. Titrations in methanolic solution were carried out in the presence of an excess of lithium hydroxide, which served to ensure deprotonation of the carboxylates under study, and a constant medium (ionic strength, proton activity) throughout the titrations. As in previous studies on related systems (i.e. Eu_2_⋅**2**, Scheme 1), Eu_2_⋅**1** displayed strong selectivity for isophthalate at 16,660 m^−1^ in the presence of 1 mm lithium hydroxide, compared with 24,600 m^−1^ in pure methanolic solution. Not only does this provide evidence of competitive binding by hydroxide, but the value in the absence of hydroxide is five times lower than that determined for Eu_2_⋅**2**.^31^ To investigate this further, we studied the binding of phthalate and terephthalate, determining the binding constants in methanol in the presence of 1 mm lithium hydroxide. With values for *K*_phthalate_ and *K*_terephthalate_ of 50,900 m^−1^ and 29,300 m^−1^, respectively, compared to a *K*_isophthalate_ value of 16,660 m^−1^, we observed that the binding strength of the three phthalate isomers to Eu_2_⋅**1** are dramatically different from those previously determined for Eu_2_⋅**2**. For the system studied here (Eu_2_⋅**1**), bidentate binding by phthalate is more favourable than binding by the bridging guests. Furthermore, in this combination of solvent, proton affinity and host, terephthalate is a better bridging guest than isophthalate. This result is completely opposite to our previous findings for Eu_2_⋅**2**.^27^ This implies that even small changes in the nature of the bridging unit, such as exchanging a remote nitro group for an amino group, can lead to dramatic changes in binding affinity. Such changes might result from changes in solvent order, or conceivably through changes to the lipophilicity of the bridging aryl ring.

In methanolic lithium hydroxide, Eu_2_⋅**1** displayed high selectivity for isophthalate over dinicotinate, revealing that small changes in guest structure can have just as dramatic an effect as changes to the host. In this case, the explanation is likely to be more straightforward: the electron-withdrawing effect of the pyridine nitrogen is likely to decrease the electron-donating ability of the carboxylate groups.

### Establishing the effect of temperature

Measurement of the affinity constant for the binding of anions to Eu_2_⋅**1** at multiple temperatures in the presence of lithium hydroxide (Table 1) revealed further differences in behaviour. While isophthalate exhibited strong temperature dependence, benzoate showed much smaller variations with temperature, and both dinicotinate and nicotinate showed essentially no change in binding with temperature. Figure 2 shows titration isotherms for a single temperature for isophthalate, and the van′t Hoff plot for the temperatures where the *K* value was determined (for all guest ions, see the Supporting Information). In the case of isophthalate, the thermodynamic sense is clear; binding is disfavoured on enthalpic grounds but favoured entropically. This suggests competitive binding by hydroxide at the metal centres, in which displacement of hydroxide decreases the overall order in the system. Hydroxide binding might take many forms in methanolic media, and given the excess of hydroxide, it is conceivable that hydroxide ions coordinate to both metal centres. In such a case, the gain in entropy might result simply from the displacement of two hydroxide ions upon binding of isophthalate. It should be noted that hydroxide has been observed to bridge lanthanide centres and has also been implicated in the aggregation of mononuclear lanthanide complexes.^35^ However, the distance between the two metal centres in Eu_2_⋅**1** is likely to preclude such an arrangement.

**Table 1:** Affinity constants and thermodynamic parameters of 5-nitro-α,α′-bis(Eu⋅DO3Ayl)-*m*-xylene Eu_2_⋅1 for a range of anionic guest ions in methanol.^[a]^

	*T* [K]	isophthalate	dinicotinate	benzoate	nicotinate
*K*^[b]^ [m^−1^]	293	16 660	2534	421	570
	298	20 410	2753	455	571
	303	24 280	2525	569	638
	313	39 990	2639	–	579
Δ*H* [kJ mol^−1^]	–	33	–^[a]^	22	–^[c]^
Δ*S* [Jmol^−1^ K^−1^]	–	194	–^[a]^	125	–^[c]^

[a] All data were obtained in the presence of aq. LiOH⋅H_2_O (1 mm). [b] Confidence intervals for *K* can be found in the Supporting Information. [c] The change in *K* with temperature is within the error of the measurement, as such, meaningful thermodynamic parameters cannot be readily obtained.

In all of these titrations, 1 mm lithium hydroxide monohydrate was added to ensure that changes in solvent would not interfere with the measurements. The proton activity, solvent polarity, and ionic strength might interfere with the observed luminescence from the lanthanide complex to a degree that data on the binding event cannot be extracted.

Studies of isophthalate binding in aqueous media are inevitably precluded by the very low solubility of isophthalate in water. However, dinicotinate is water soluble, and binding studies on dinicotinate in water proved highly informative (Table 2). The most obvious observation is that the binding constants observed in borate-buffered saline (BBS) and 4-(2-hydroxyethyl)-1-piperazineethanesulfonic acid (HEPES) buffer are dramatically greater than those measured in methanolic lithium hydroxide. In itself, this would be suggestive of competitive binding of hydroxide. Further evidence for this hypothesis was provided by studies carried out at pH 11, where millimolar hydroxide concentrations led to competitive binding of hydroxide. In such systems, no change in lanthanide luminescence was observed with increasing dinicotinate concentration (see the Supporting Information), clearly showing that dinicotinate cannot outcompete hydroxide binding in water.

**Table 2:** Affinity constants and thermodynamic parameters of 5-nitro-α,α′-bis(Eu⋅DO3Ayl)-*m*-xylene Eu_2_⋅1 for dinicotinate at 293 K in aqueous media.^[a]^

	*T* [K]	pH 8.1 (BBS)	pH 7.5 (HEPES)	pH 7.4 (PBS)	pH 11 (H_2_O)
*K*^[b]^ [m^−1^]	293	29 830	17 730	0	0
	303	11 320	13 220	0	0
	313	9200	9650	0	0
Δ*H* [kJ mol^−1^]	–	−45.0	−23.2	–	–
Δ*S* [Jmol^−1^ K^−1^]	–	−69.5	−2.2	–	–

[a] No change in luminescence was observed upon titration in either PBS or at pH 11. [b] Confidence intervals for *K* can be found in the Supporting Information.

From the data in Table 2, it is immediately clear that the affinity constant of Eu_2_⋅**1** for dinicotinate in water has a very different temperature dependence to the same system in methanolic lithium hydroxide (for the van′t Hoff plots, see the Supporting Information). In this case, the affinity for dinicotinate decreases with increasing temperature in both BBS and HEPES buffer. In both buffers, the binding is enthalpy driven and disfavoured on entropic grounds. Furthermore, the difference between these parameters in BBS and HEPES buffer strongly suggests that HEPES buffer is a competitive buffer in this system. Such effects have been observed for the pyridine-bridged system, where HEPES is a more competitive ion than phosphate.^28^

### Self-assembly in biological mimics

No binding was observed in phosphate-buffered saline (PBS), presenting the argument that phosphate also outcompetes dinicotinate. This is in contrast with our earlier observations, which showed no effect of phosphate upon the overall affinity.^28^ It is worth noting that the inner sphere hydration (where *q* is the number of inner sphere solvent molecules)^36^ of Eu_2_⋅**1** is significantly greater than that of Eu_2_⋅**2** (*q*(Eu_2_⋅**1**)=1.2 and *q*(Eu_2_⋅**2**)=0.8, see Ref. 34). As such, Eu_2_⋅**1** would be expected to display a greater affinity for phosphate, since phosphate can act as a bidentate donor at one of the lanthanide centres. This assumption is supported by the high affinity of Eu_2_⋅**1** for phthalate, which is unlikely to bridge between the two lanthanide centres. This could explain why no sign of host–guest interactions was observed between dinicotinate and Eu_2_⋅**1** in PBS (Table 2).

The data in Table 1 clearly shows that the dinicotinate/Eu_2_⋅**1** binding partners would be unsuitable for studies in biological systems, where phosphate will outcompete the association between the two. To investigate whether the self-assembly with Eu_2_⋅**1** could be affected by other endogenous ions than phosphate, we studied the binding of citrate and lactate in methanol, and we were not able to observe any binding. So to investigate the effect of arterial concentrations of lactate (2 mm), pyruvate (0.3 mm), and citrate (0.01 m),^37^,^38^ Eu_2_⋅**1** was titrated with dinicotinate in BBS at pH 8.2 in the presence of arterial concentrations of these. The results show that all three ions exhibit competitive binding. The association between Eu_2_⋅**1** and dinicotinate has an affinity constant (*K*_BBS_) of 29,830 m^−1^ (Table 2), the affinity is decreased by a factor of ten in the presence of lactate (*K*_BBS lactate_=3,550 m^−1^), while citrate decreased the affinity by a factor of 20 (*K*_BBS citrate_=1,640 m^−1^). In the presence of biological concentrations of pyruvate, no binding is observed. In contrast to Eu_2_⋅**2**, the solution structure of Eu_2_⋅**1** allows for competitive binding of ligands that permits bidentate binding to a single lanthanide centre such as phosphate, phthalate, and pyruvate.

### Changes in the luminescence spectra

The steady-state luminescence spectra of the complexes change dramatically between solvent systems (Figure 3) as a consequence of changes in the local environment at the europium centres. Not only does the relative intensity of the bands change, as would be expected given the hypersensitivity of the ^5^D_0_–^7^F_2_ transition, but the fine structure of individual bands changes dramatically. This is clear evidence for different local symmetries at the metal centres.

Study of the steady-state spectra before and after addition of dicarboxylates also reveals that there are significant changes to the excitation spectra (Figure 1). The spectra show that the observed increases in luminescence intensity can be attributed to the additional absorption cross-section of the dinicotinate and isophthalate chromophores, meaning that luminescence is sensitised by both host and guest. This is clearly the dominant effect, and changes to hydration and removal of other non- radiative quenching pathways for the lanthanide excited state are of negligible importance in establishing the affinity of host for guest.

**Figure 4:**
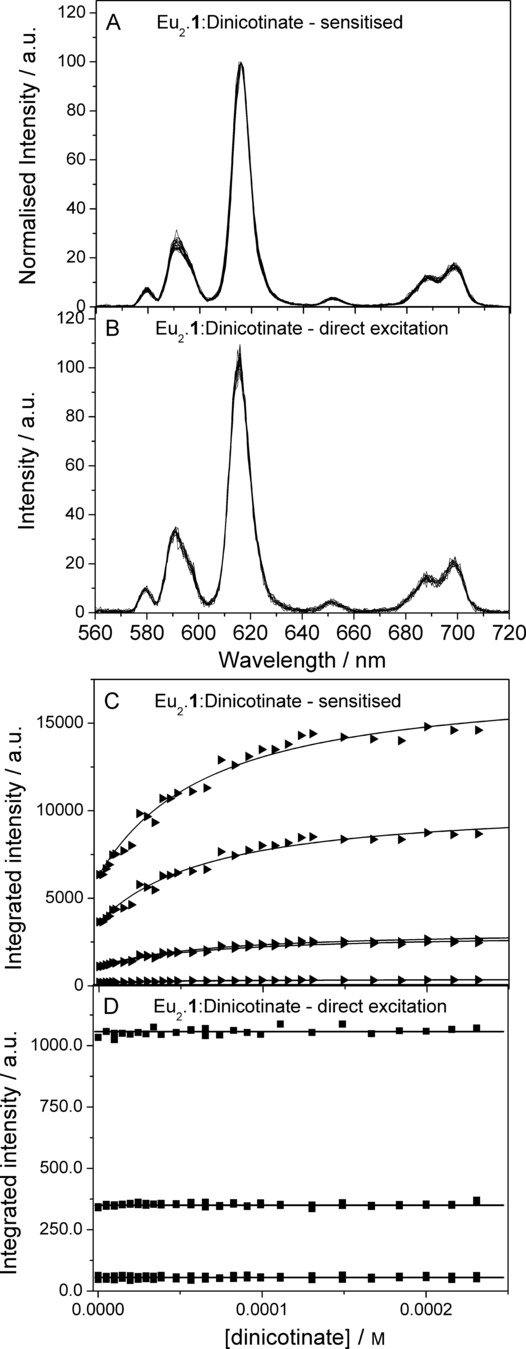
Data from a titration of 5-nitro-α,α′-bis(Eu⋅DO3Ayl)-*m*-xylene (Eu_2_⋅1; 0.01 mm) with dinicotinate (1 mm) in 20 mm HEPES buffer at pH 7.4. A) Normalised emission spectra measured following 240 nm excitation; all spectra from a full titration are included. B) Raw-data emission spectra measured following 395 nm excitation from the same titration depicted in panel A. C) Titration isotherm and fit to the data obtained following 240 nm excitation. D) Titration isotherm from data obtained following 395 nm excitation; horizontal lines have been added as a guide.

To further investigate contributions leading to the intensity increase, titrations were performed using direct (395 nm, ^7^F_5_–^5^L_6_) and sensitised excitation. Figure 4 shows the normalised spectra following from sensitised excitation (Figure 4 A); this is done in order to show that no changes in either the band shape or intensity can be seen (for the raw data, see the Supporting Information). Following direct excitation, the spectra do not change as the titration progresses (Figure 4 B), which clearly indicates that the overall quantum yield of emission—the sum of the seven individual contributions from the seven different possible transitions from ^5^D_0_—does not change as the titration progresses. This is counterintuitive as it would be expected that the binding of anions occurs with displacement of water, lowering the number of quenching X−H oscillators close to the lanthanide centres, increasing the quantum yield of emission. The titration isotherms plotted in Figure 4 C–D show that this is clearly not the case.

For most of the titrations performed in this study, a small change can be seen in the spectra following direct excitation at the beginning and the end of the titrations. Closer scrutiny of the excitation spectra at these points in the titrations (Figure 1) show that this is due to a tail of absorption from the guest chromophore, not an effect in the direct excitation pathway.

### Time-resolved luminescence studies

Measurement of the luminescence lifetimes of the complexes and assemblies in solution (Table 3) yielded further information about the binding event. It is clear that the luminescence lifetimes in a variety of aqueous media change only slightly (and within the standard margin of error) upon addition of isophthalate, implying that any change in the inner solvation sphere as a consequence of binding is remarkably limited. In methanolic solution, the lifetimes in the absence and presence of all potential guests are also the same within error, suggesting that (in this case too) the guest supplements solvent molecules in the inner coordination sphere rather than displacing them. In all systems, luminescence lifetimes were found to be invariant with temperature in the range from 283 to 333 K.

**Table 3:** Luminescence lifetimes (τ) of 5-nitro-α,α′-bis(Eu⋅DO3Ayl)-*m*-xylene Eu_2_⋅1 in various solvents and with a variety of guest ions.

Guest ion	τ^[a]^ [ms]			
	pH 8.1 (BBS)	pH 7.5 (HEPES)	pH 7.4 (PBS)	MeOH (1 mm LiOH)
none	0.63	0.53	0.61	1.07
dinicotinate	0.77	0.64	0.69	1.15
isophthalate	–	–	–	1.15
nicotinate	–	–	–	1.07
benzoate	–	–	–	1.09

[a] Error values for τ are ±5 % or less.

Indeed, calculation of the number of inner sphere solvent molecules (*q*) using the modified Horrocks equation^36^,^39^ (Table 4), emphasises this point about coordination chemistry. It is clear that the overall solvation at the lanthanide centres is initially lower than might be expected, and consistent with one water molecule per lanthanide in aqueous media. In these systems, the lipophilicity of the bridging *m*-xylyl group would be expected to mediate against the close proximity of two water molecules despite the heptadentate nature of both binding pockets. However, favourable interactions between this bridging group and the aryl carboxylate guests might also be expected and would indeed enhance the efficiency of binding. The same phenomenon is observed in methanolic solution. pH does have a significant effect upon both the lifetime in aqueous solution and the value of *q*; the lifetime increases and *q* is decreased as pH increases as a direct consequence of deprotonation of bound water at the metal centre. In other systems, variation in *q* with pH has been assigned to bridging oxo or hydroxo groups that link two lanthanides together.^35^ While it would be appealing to use a similar explanation in this case, there is no other evidence for bridging by hydroxide.

**Table 4:** Variation in luminescence lifetimes (τ) and the number of inner sphere solvent molecules (*q*) of 5-nitro-α,α′-bis(Eu⋅DO3Ayl)-*m*-xylene Eu_2_⋅1 with pH in aqueous media.

Medium	pH	pD	τ^[a]^ [ms]		*q*^[b]^
			H_2_O	D_2_O	
water	2.0	2.1	0.53	1.36	1.1
	11.3	11.5	0.65	1.63	0.8
	12.5	12.8	0.64	1.57	0.8
methanol	1 mM LiOH		1.07	1.55	0.8

[a] Error values for τ are 5 % or less. [b] Error values for *q* are within 0.5; for the definition of *q* and calculation method, see Ref. 22.

## Conclusion

It is clear from these results that the thermodynamics of self-assembly between binuclear lanthanide complexes and dianions are finely balanced, and far from the simple picture where lanthanide-centred interactions are dominated by coloumbic forces. In the system studied here, the role of solvent remains key to overall behaviour. Furthermore, competitive binding by a range of anions can change the observed affinity.

We find that these binuclear lanthanide complex hosts constitute a single supramolecular binding pocket,^40^ where the nature of the guest determines the number of favourable interactions that can be exploited: hydrophobic interactions with the aryl bridge and one or two interactions to a lanthanide centre. These systems are simple when seen from the perspective of host–guest chemistry. However, taken together with our other studies on related systems, it is becoming increasingly clear that small variations in the molecular structure of either guest or host can have dramatic effects on both affinity and selectivity.

Additionally, it is particularly notable that two closely related systems display a remarkably different response to the presence of phosphate. This is potentially of vital importance when considering whether self-assembly can be exploited in vivo, where competition with phosphate must be ruled out. Such studies would also require a detailed understanding of competitive binding by endogenous anions.

## Experimental Section

5-Nitro-α,α′-bis(Eu⋅DO3Ayl)-*m*-xylene (Eu_2_⋅**1**) was prepared as previously described.^31^ The salt content was determined using elemental analysis, while the europium content was determined using inductively coupled plasma mass spectrometry (ICP-MS). All concentrations were corrected appropriately. Lithium benzoate (>99 %), lithium hydroxide monohydrate (99.95 %), nicotinic acid (>99.5 %), and isophthalic acid (99 %) were purchased from Sigma–Aldrich. Dinicotinic acid (pyridine-3,5-dicarboxylic acid, 98 %) was purchased from Alfa Aesar, and the purity was verified by elemental analysis. Ultrapure water from a MilliQ system and HPLC grade methanol were used as solvents. Buffers were prepared from commercial tablets (PBS, BBS) or from the pure form with KCl and NaCl (HEPES), all purchased from Sigma–Aldrich. Emission spectra were recorded using a Varian Cary Eclipse and a Horiba Fluorolog-3 spectrometer. Absorption spectra were recorded using a PerkinElmer Lambda1050 spectrometer. Temperature control was achieved using a Cary single-cell Peltier controller, while monitoring the temperature of the cuvette holder continuously and the solution intermittently. The temperature was maintained at the target temperature ±0.1 °C.

Solutions were prepared in volumetric flasks, and the compound mass was determined to three significant figures. A single buffer or solvent stock solution was prepared and used to prepare all other solutions in order to ensure a uniform solvent composition. A ∼0.01 mm stock solution of Eu_2_⋅**1** was prepared for each solvent system, and this was used to prepare the 1 mm titrant solution. The pH of aqueous buffer solutions were adjusted to be identical within 0.01 pH unit.

Titrations were performed by placing 1 or 2 mL of Eu_2_⋅**1** solution in a 2 mm and 10 mm pathway cuvette using volume displacement pipettes. The titrant solution was added using volume displacement pipettes, the cuvette was shaken to mix the solution, and then the cuvette was briefly allowed to equilibrate in the Peltier-controlled cuvette holder. The tiny aliquot added in combination with the small temperature difference (Δ*T*<20 °C) did not alter the solution temperature to any measurable extent. Excitation spectra were recorded at the beginning and the end of each titration, while emission spectra were recorded at each data point. The individual bands in the emission spectra were integrated and plotted against titrant concentration to generate binding isotherms. These data were fitted using DynaFit 4 as previously reported,^30^,^31^,^41^,^42^ to determine the affinity constant (*K* in m^−1^), defined by Equation (1), where X represents a guest: benzoate, isophthalate, nicotinate, or dinicotinate.

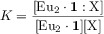
1

A van′t Hoff plot of −*R*ln*K* against *T*^−1^ allowed determination of the enthalpic and entropic contributions to the binding as the slope and the intercept, respectively [Equation (2)].

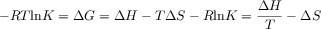
2

Spectra and isotherms from all titrations can be found in the Supporting Information, along with the Arrhenius plots for dinicotinate in BBS and HEPES buffer.

## Supporting Information

As a service to our authors and readers, this journal provides supporting information supplied by the authors. Such materials are peer reviewed and may be re-organized for online delivery, but are not copy-edited or typeset. Technical support issues arising from supporting information (other than missing files) should be addressed to the authors.
