# Supplementary material for: Thermodynamics of Self-Assembly of Dicarboxylate Ions with Binuclear Lanthanide Complexes
Source: ChemistryOpen. 2015 Jun 25;4(4):509–15. doi: 10.1002/open.201500060 (PMC4603413; doi:10.1002/open.201500060)

ChemistryOPEN

Supporting Information

© 2015 The Authors. Published by Wiley-VCH Verlag GmbH & Co. KGaA, Weinheim

Thermodynamics of Self-Assembly of Dicarboxylate Ions with Binuclear Lanthanide Complexes

Thomas Just Sørensen,*[a, b] Leila R. Hill,[b] and Stephen Faulkner*[b]

open_201500060_sm_miscellaneous_information.pdf

Table of contents

Table of contents 1

General information 2

Binding constants with errors 2

Titrations – water 3

- 1 mM LiOH in H_2O –isophthalate – 25 °C 3
- 1 mM LiOH in H_2O –dinicotinate – 25 °C 3
- PBS pH 7.4 –dinicotinate – 25 °C 3
- Dinicotinate in HEPES van't Hoff plot 4
- HEPES pH 7.5 –dinicotinate – 10 °C 4
- HEPES pH 7.5 –dinicotinate – 20 °C 4
- HEPES pH 7.5 –dinicotinate – 30 °C 5
- HEPES pH 7.5 –dinicotinate – 40 °C 5
- Dinicotinate in BBS van't Hoff plot 5
- BBS pH 8.1 –dinicotinate – 10 °C 6
- BBS pH 8.1 –dinicotinate – 20 °C 6
- BBS pH 8.1 –dinicotinate – 30 °C 6
- BBS pH 8.1 –dinicotinate – 40 °C 7
- BBS pH 8.1 –dinicotinate – 25 °C – with 2 mM lactate 7
- BBS pH 8.1 –dinicotinate – 25 °C – with 10^{-5} M citrate 7
- BBS pH 8.1 –dinicotinate – 25 °C – with approx. 0.5 mM pyruvate 8

Titrations – Methanol 9

- Benzoate in Methanol van't Hoff plot 9
- Dinicotinate in Methanol van't Hoff plot 9
- Nicotinate in Methanol van't Hoff plot 10
- 1 mM LiOH in methanol –benzoate – 20 °C 10
- 1 mM LiOH in methanol –benzoate – 25 °C 10
- 1 mM LiOH in methanol –benzoate – 30 °C 11
- 1 mM LiOH in methanol –benzoate – 40 °C 11
- Methanol –isophthalate – 20 °C 11
- 1 mM LiOH in methanol –isophthalate – 20 °C 12
- 1 mM LiOH in methanol –isophthalate – 25 °C 12
- 1 mM LiOH in methanol –isophthalate – 30 °C 12
- 1 mM LiOH in methanol –isophthalate – 40 °C 13
- 1 mM LiOH in methanol –nicotinate – 20 °C 13
- 1 mM LiOH in methanol –nicotinate – 25 °C 13
- 1 mM LiOH in methanol –nicotinate – 30 °C 14
- 1 mM LiOH in methanol –nicotinate – 40 °C 14
- 1 mM LiOH in methanol –dinicotinate – 20 °C 14
- 1 mM LiOH in methanol –dinicotinate – 25 °C 15
- 1 mM LiOH in methanol –dinicotinate – 30 °C 15
- 1 mM LiOH in methanol –dinicotinate – 40 °C 15
- 1 mM LiOH in methanol – phthalate – 20 °C 16
- 1 mM LiOH in methanol – terephthalate – 20 °C 16

Luminescence decay profiles 17

- Pure H_2O and D_2O 17
- Titrations in buffers 17
- Methanolic solution 19

General information

All spectra were recorded following 240 nm excitation. Where binding is observed the binding isotherm and the best fit to the data is given as an image generated by Dynafit®. Where no binding is observed the data was not fitted.

Binding constants with errors

Table S1. Affinity Constants of 5-nitro-α,α'-bis(Eu.DO3Ayl)-m-xylene Eu_2.**1** for a range of anionic guests in methanol.

	T / K	isophthalate	confidence interval[b]	dinicotinate	confidence interval[b]	benzoate	standard error[b]	nicotinate	standard error[b]
K[b] / M^{-1}	293	16660	16510 - 16800	2534	2528 - 2541	421	21	570	10
	298	20410	20290 - 20530	2753	2746 - 2760	455	9	571	40
	303	24280	24160 - 24400	2525	2517 - 2535	569	8	638	8
	313	39990	39640 - 40180	2639	2635 - 2644	(112)[c]	81	579	9

All data was obtained in the presence of 1 mM LiOH. [a] For these systems, the change in K with temperature is within the error of the measurement. As such meaningful thermodynamic parameters cannot readily be obtained. [b] The error of K as determined in the fitting procedure. [c] Fit not converged.

Table S2. Affinity constants of 5-nitro-α,α'-bis(Eu.DO3Ayl)-m-xylene Eu_2.**1** for dinicotinate at 293K in aqueous media.

		BBS	confidence interval[b]	HEPES	confidence interval[b]	PBS	confidence interval[b]	H_2O	confidence interval[b]
	T / K	pH 8.1		pH 7.5		pH 7.4		pH11	
K[b] / M^{-1}	293	29830	28770 - 30940	17730	17400 - 18060	0	-	0	-
	303	11320	11100 - 11540	13220	13060 - 13390	0	-	0	-
	313	9200	9050 - 9360	9650	9470 - 9820	0	-	0	-

All data was obtained in the presence of 1 mM LiOH. [a] For these systems, the change in K with temperature is within the error of the measurement. As such meaningful thermodynamic parameters cannot readily be obtained. [b] The error of K as determined in the fitting procedure.

Table S3. Affinity constants of 5-nitro-α,α'-bis(Eu.DO3Ayl)-m-xylene Eu_2.**1** and various guest in methanol at 293 K.

	Phthalate	confidence interval[b]	isophthalate	confidence interval[b]	terephthalate	confidence interval[b]	citrate	lactate
K[b] / M^{-1}	50900	49110 - 52820	16660	16510 - 16800	29300	28640 - 29960		

All data was obtained in the presence of 1 mM LiOH. [a] For these systems, the change in K with temperature is within the error of the measurement. As such meaningful thermodynamic parameters cannot readily be obtained. [b] The error of K as determined in the fitting procedure.

Titrations – water

1 mM LiOH in H_2O –isophthalate – 25 °C

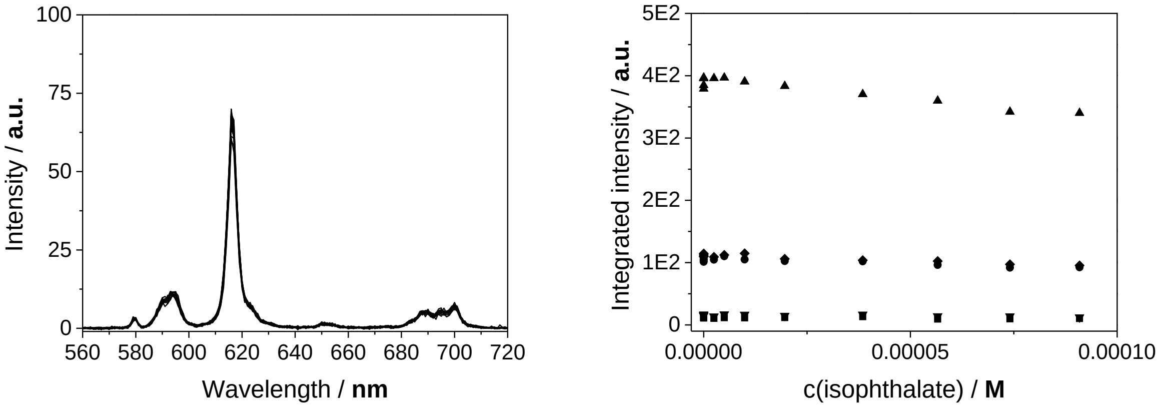

1 mM LiOH in H_2O –dinicotinate – 25 °C

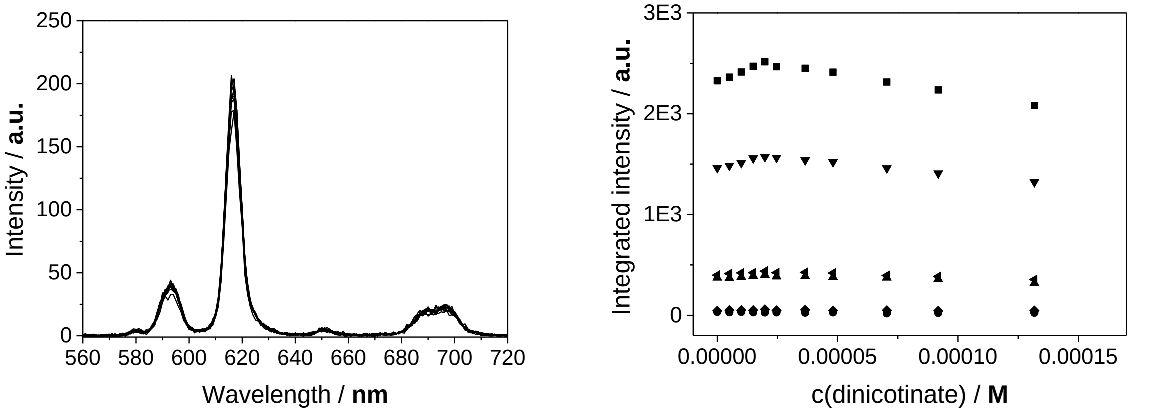

PBS pH 7.4 –dinicotinate – 25 °C

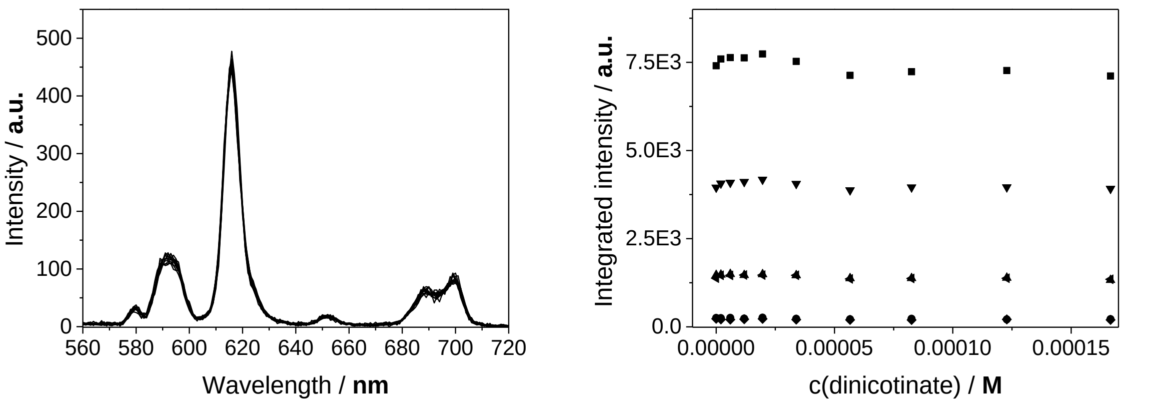

Dinicotinate in HEPES van't Hoff plot

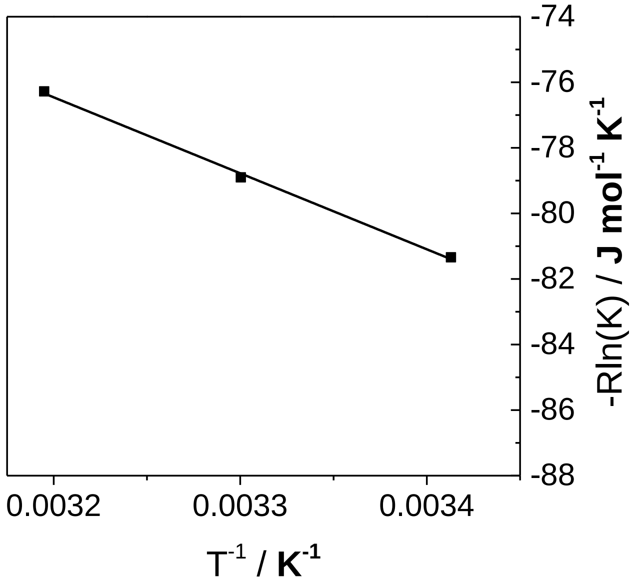

HEPES pH 7.5 –dinicotinate – 10 °C

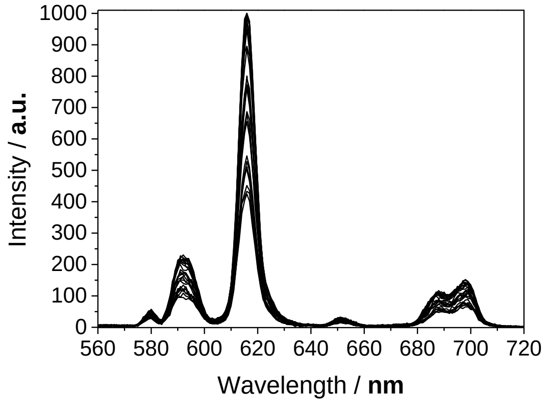

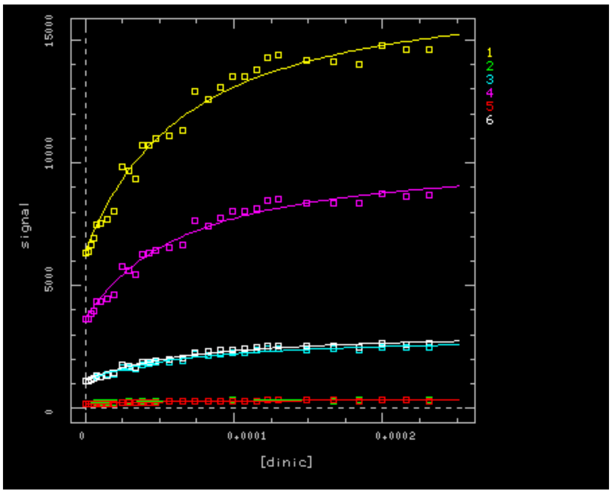

HEPES pH 7.5 –dinicotinate – 20 °C

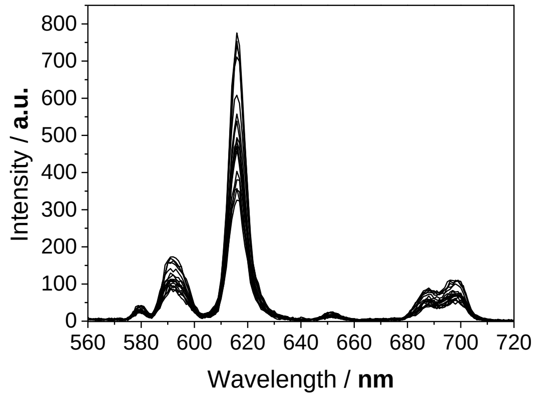

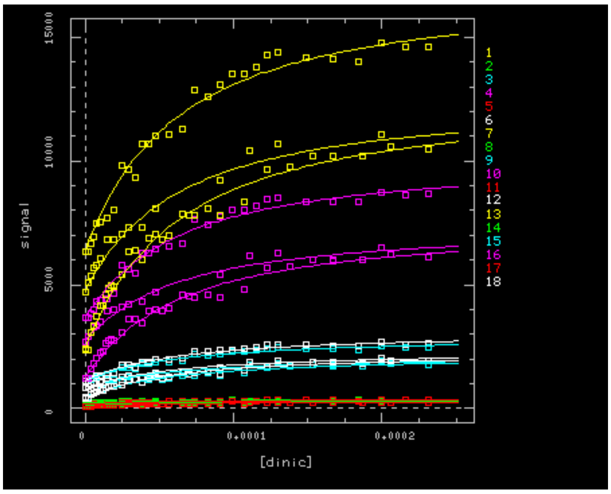

HEPES pH 7.5 –dinicotinate – 30 °C

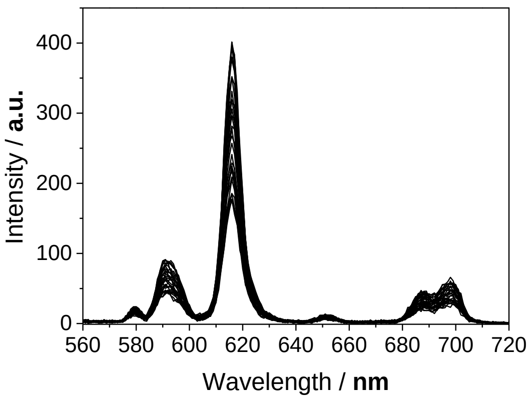

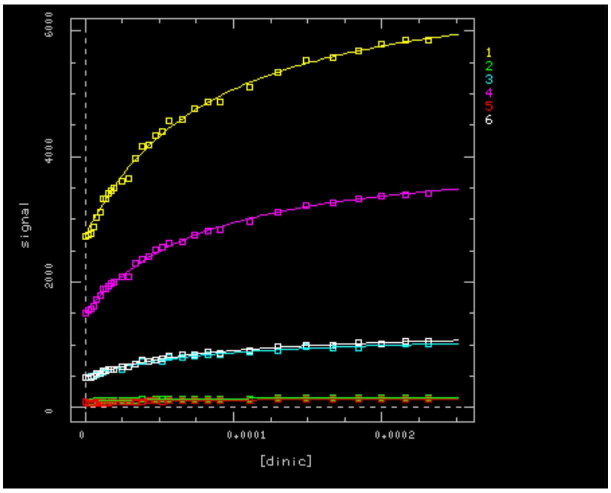

HEPES pH 7.5 –dinicotinate – 40 °C

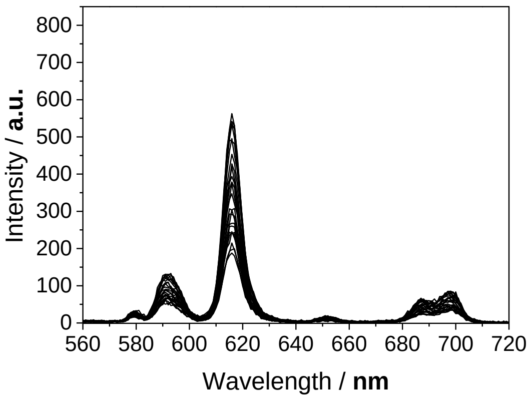

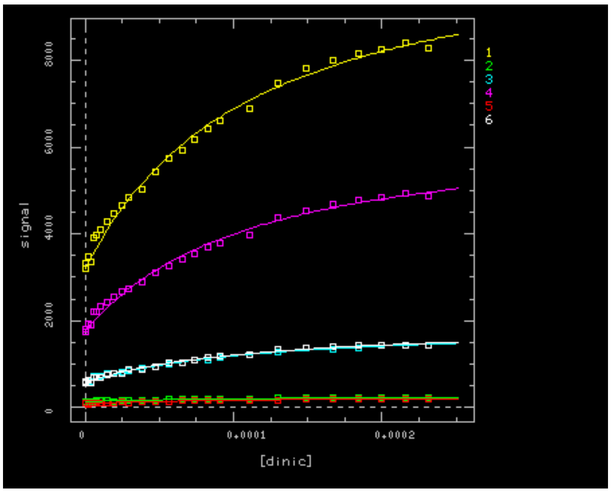

Dinicotinate in BBS van't Hoff plot

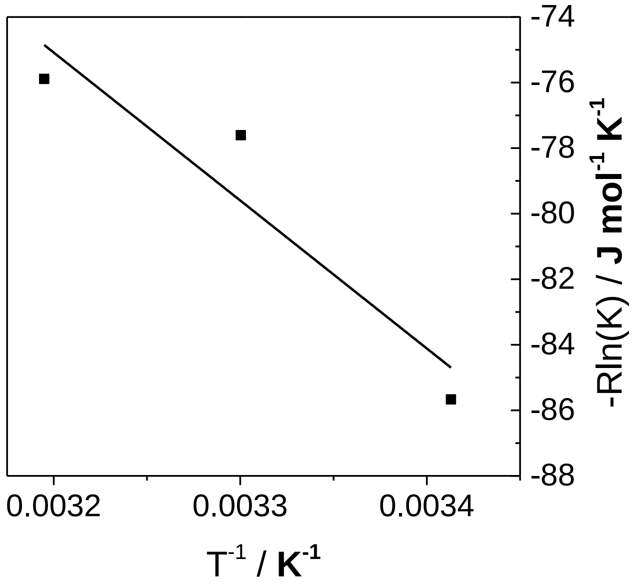

BBS pH 8.1 –dinicotinate – 10 °C

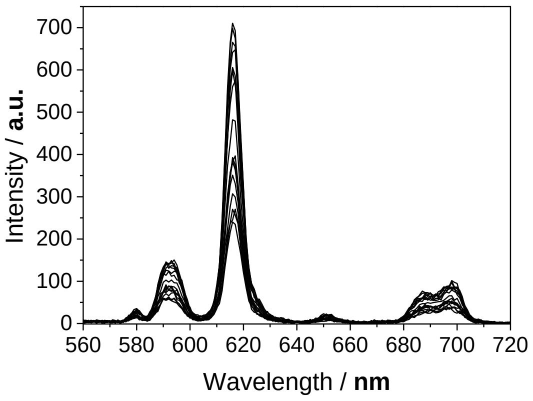

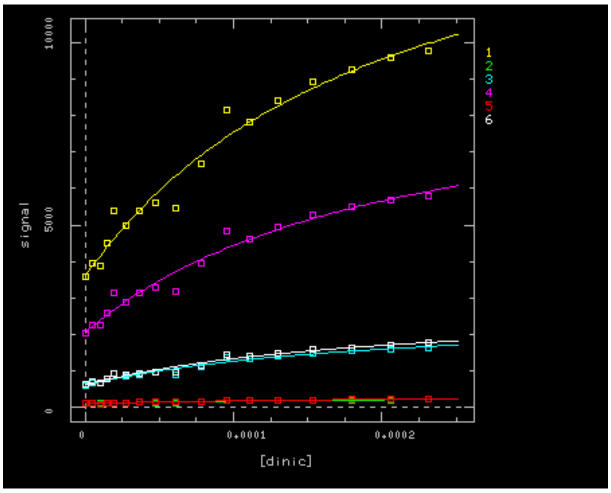

BBS pH 8.1 –dinicotinate – 20 °C

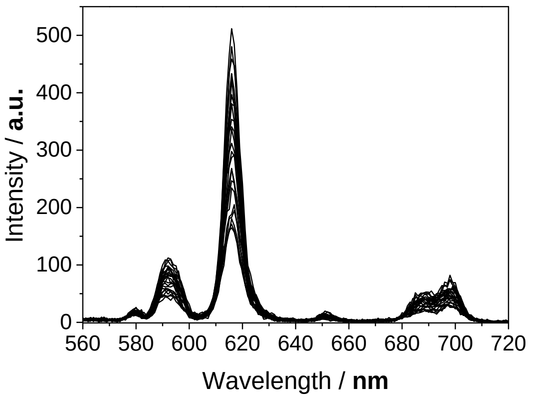

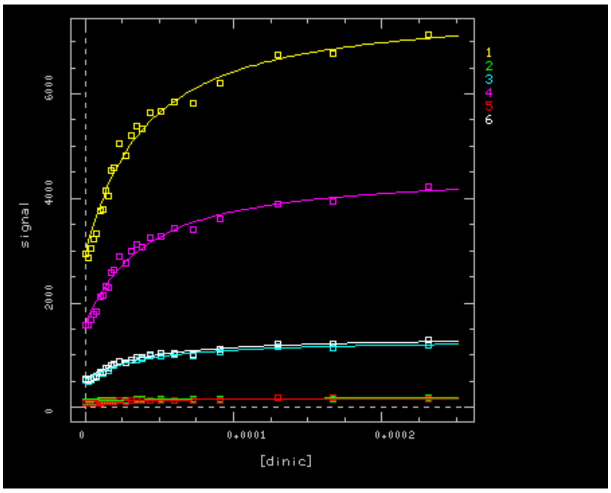

BBS pH 8.1 –dinicotinate – 30 °C

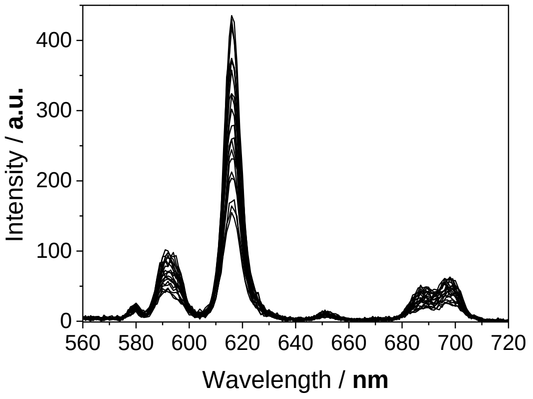

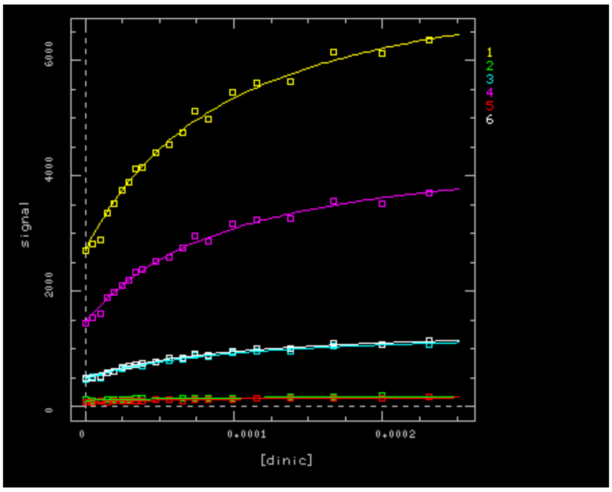

BBS pH 8.1 –dinicotinate – 40 °C

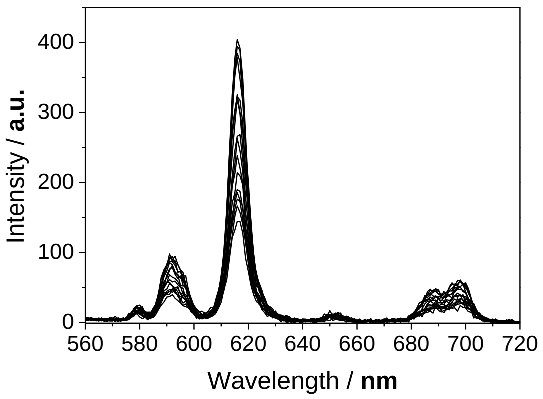

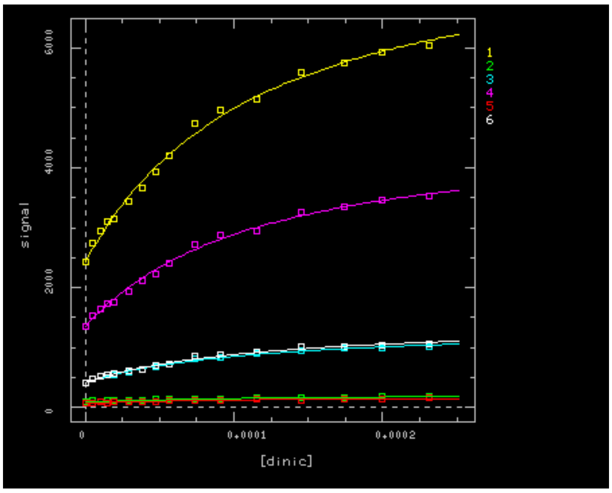

BBS pH 8.1 –dinicotinate – 25 °C – with 2 mM lactate

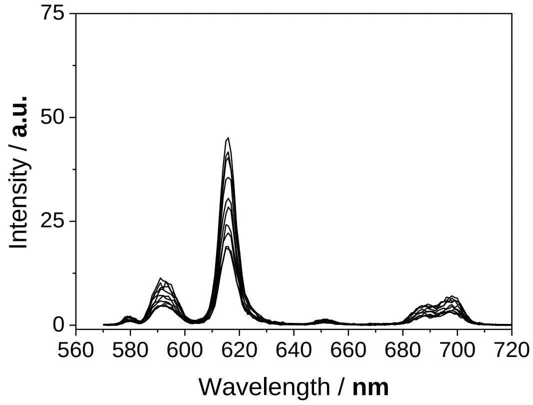

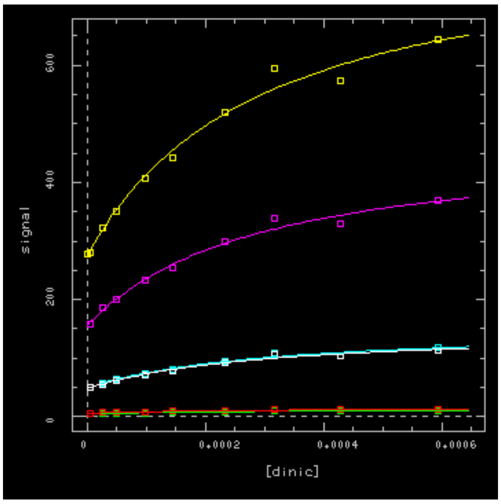

BBS pH 8.1 –dinicotinate – 25 °C – with 10^{-5} M citrate

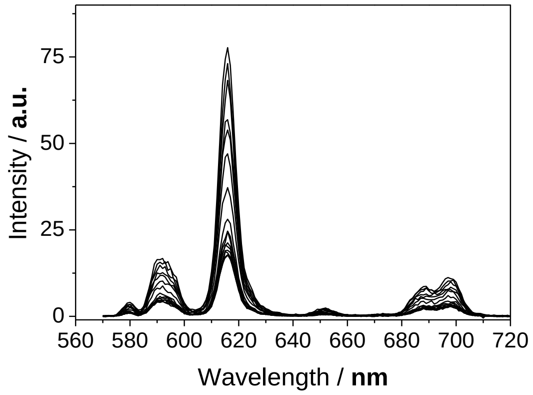

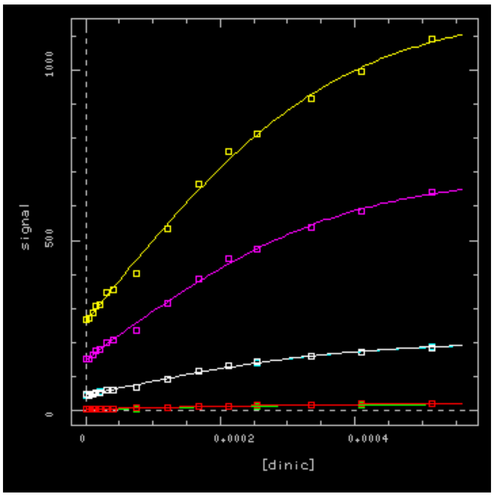

BBS pH 8.1 –dinicotinate – 25 °C – with approx. 0.5 mM pyruvate

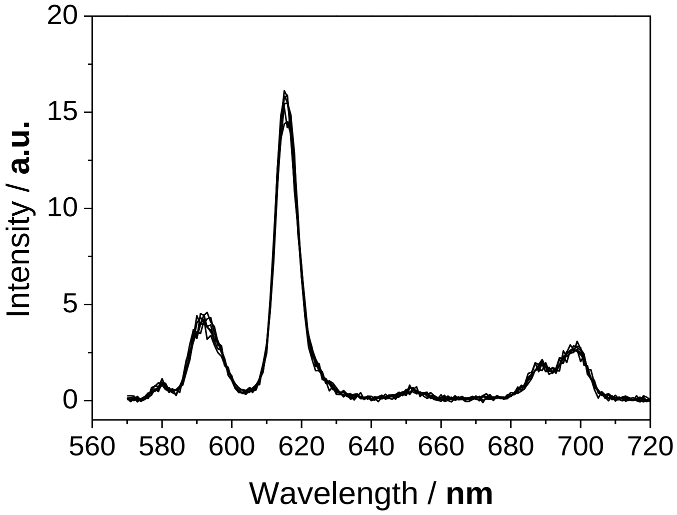

Titrations – Methanol

Benzoate in Methanol van't Hoff plot

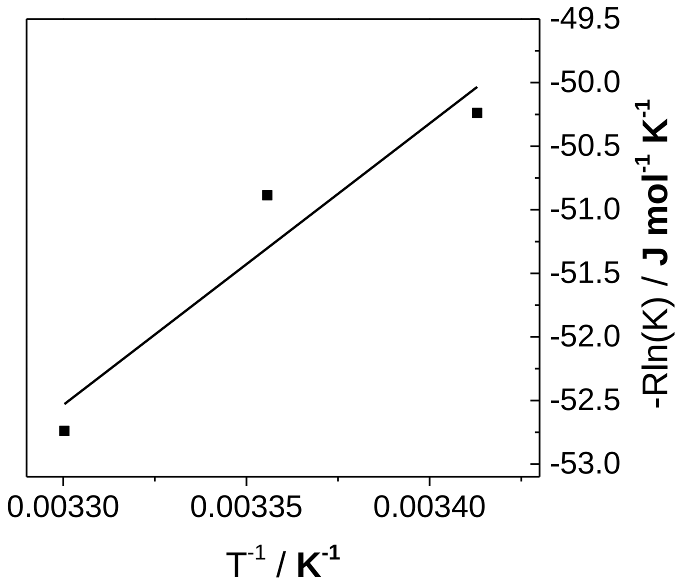

Dinicotinate in Methanol van't Hoff plot

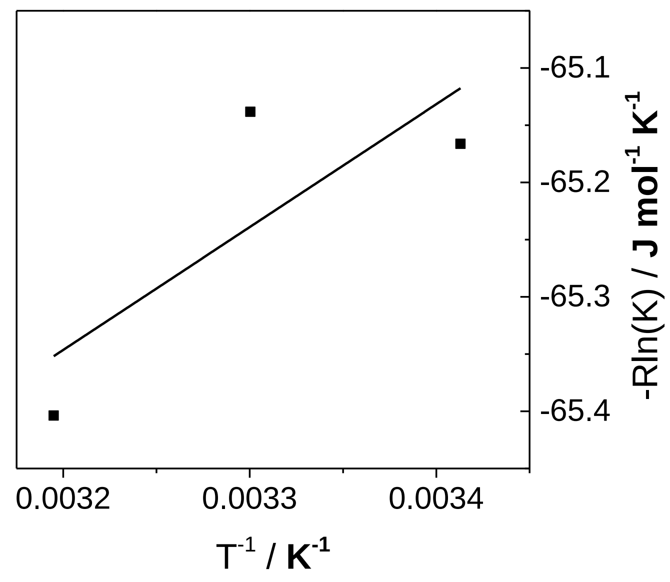

Nicotinate in Methanol van't Hoff plot

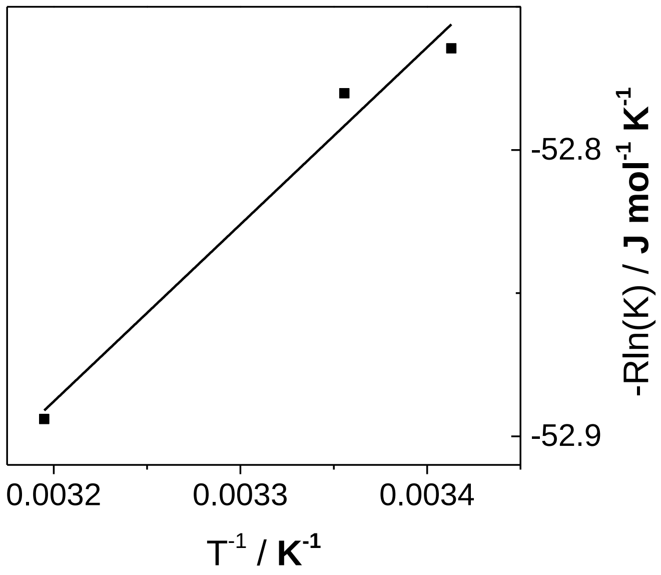

1 mM LiOH in methanol –benzoate – 20 °C

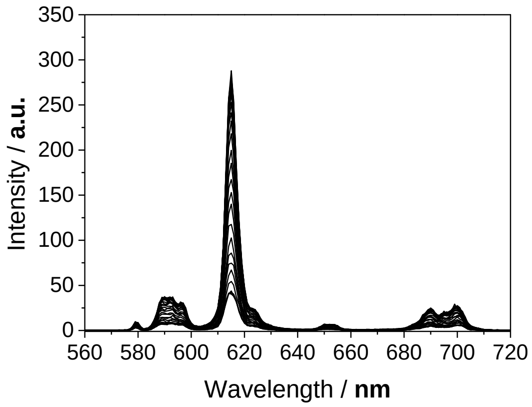

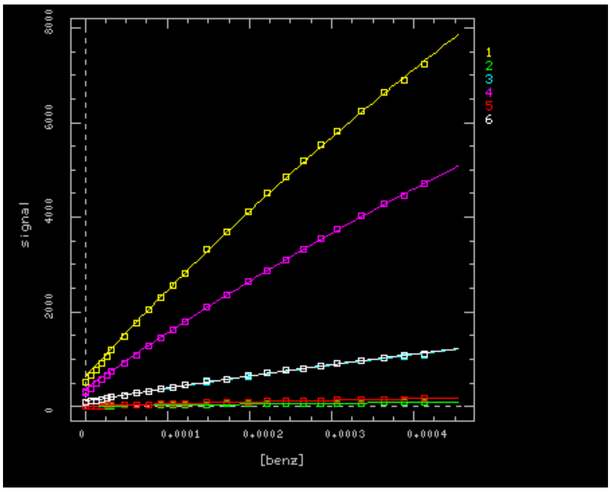

1 mM LiOH in methanol –benzoate – 25 °C

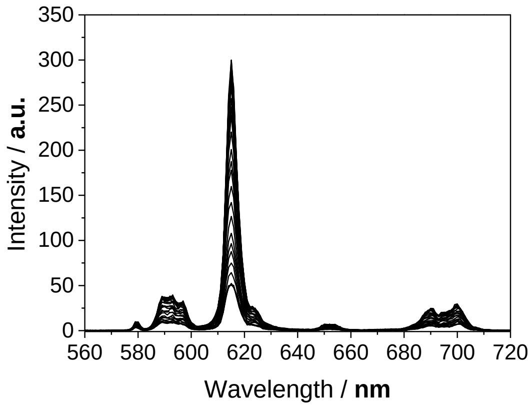

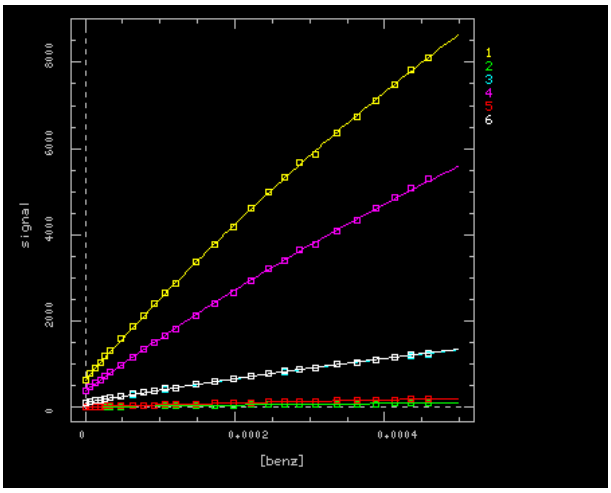

1 mM LiOH in methanol –benzoate – 30 °C

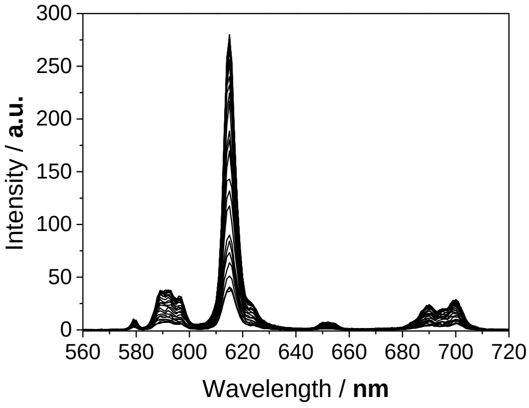

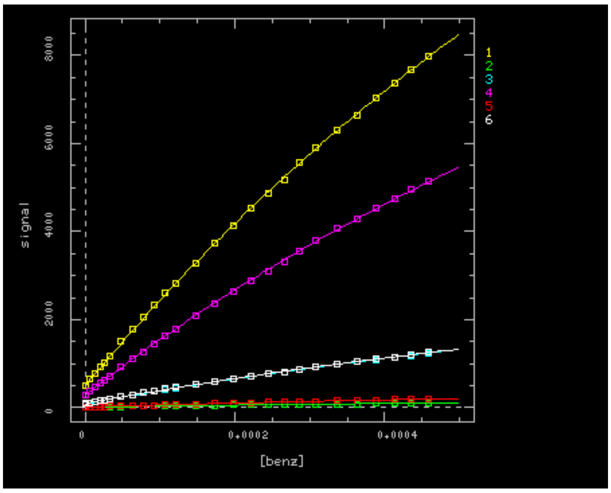

1 mM LiOH in methanol –benzoate – 40 °C

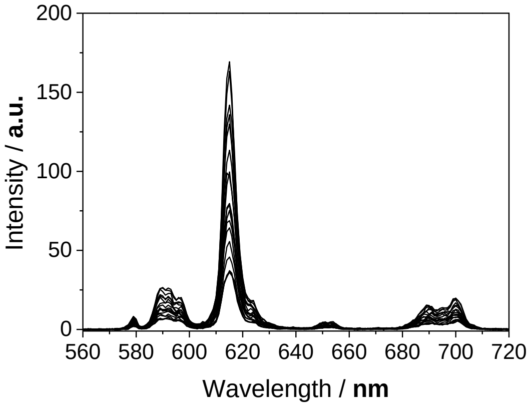

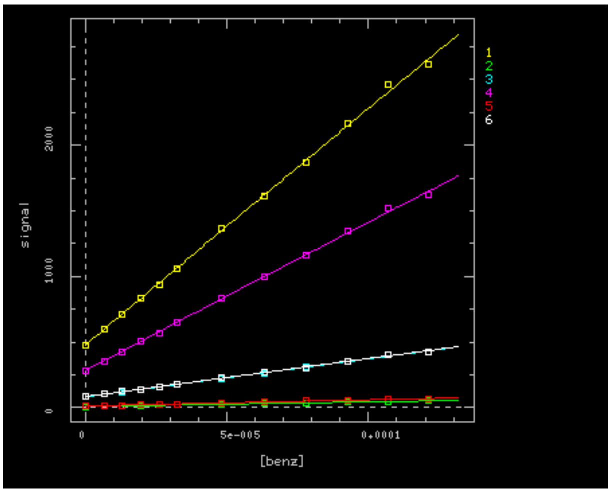

Methanol –isophthalate – 20 °C

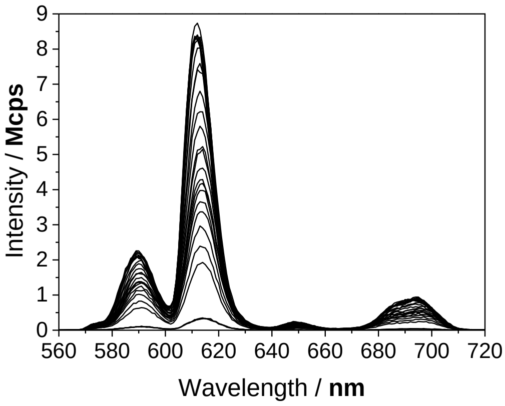

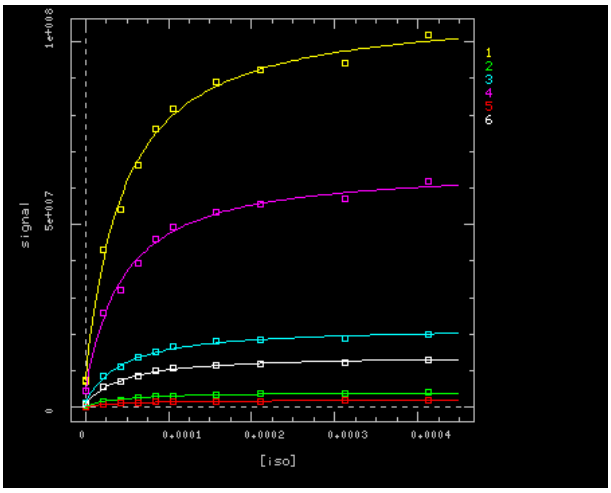

1 mM LiOH in methanol –isophthalate – 20 °C

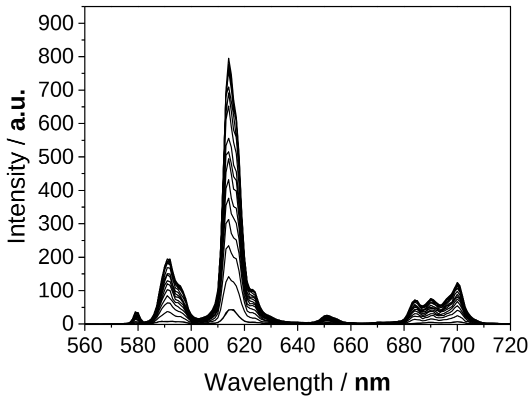

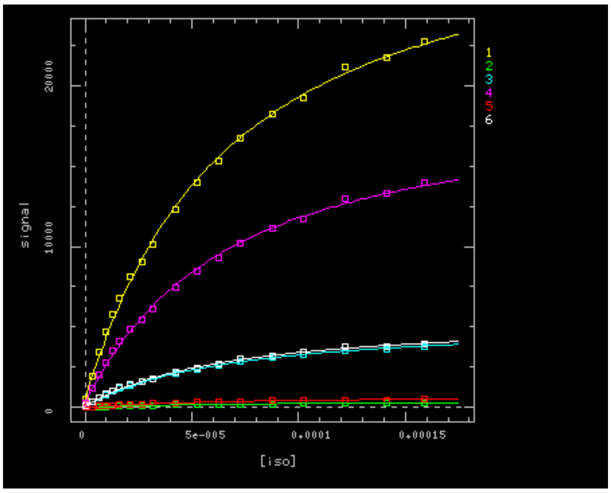

1 mM LiOH in methanol –isophthalate – 25 °C

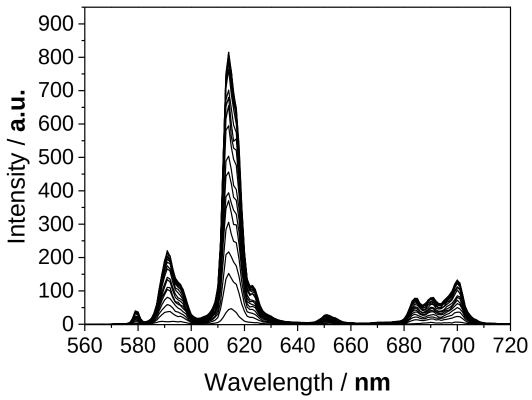

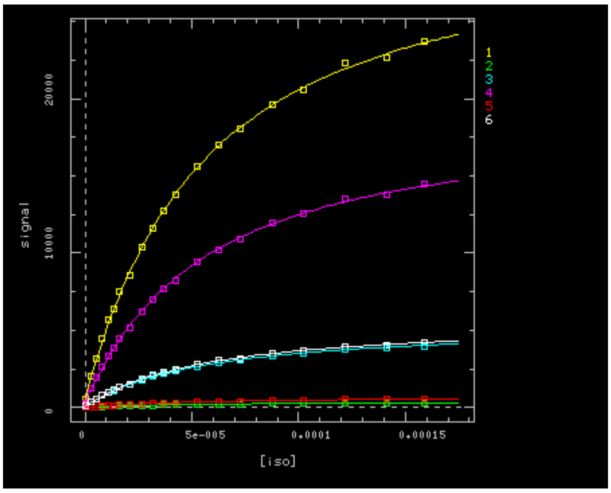

1 mM LiOH in methanol –isophthalate – 30 °C

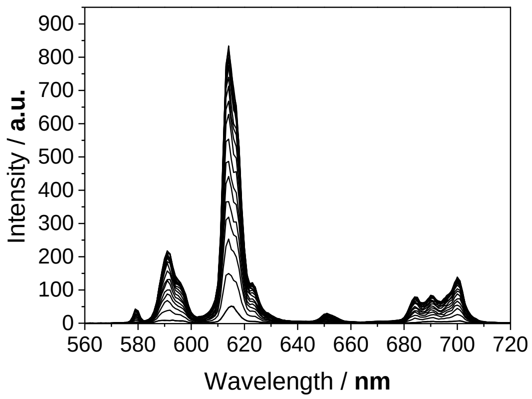

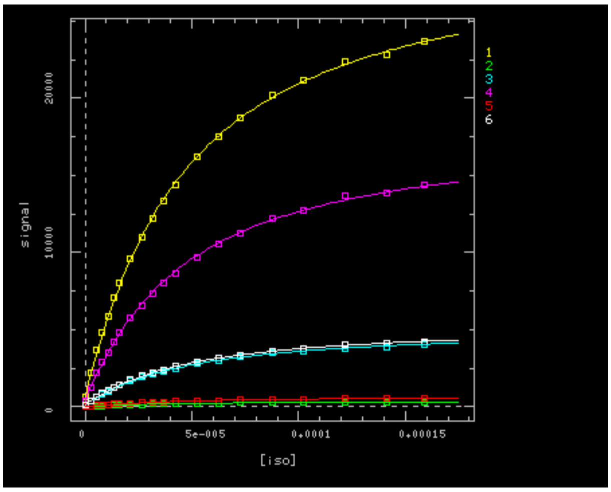

1 mM LiOH in methanol –isophthalate – 40 °C

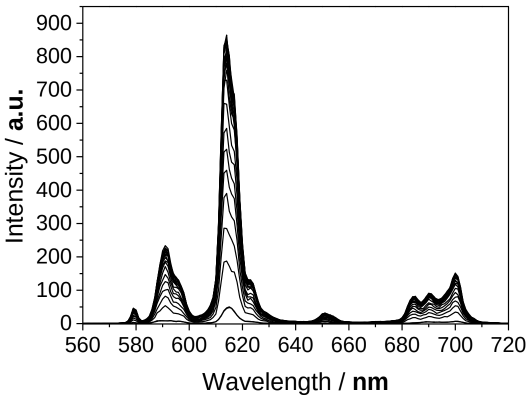

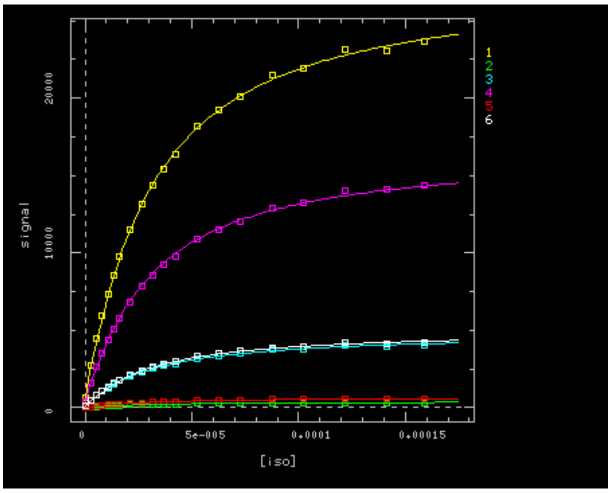

1 mM LiOH in methanol –nicotinate – 20 °C

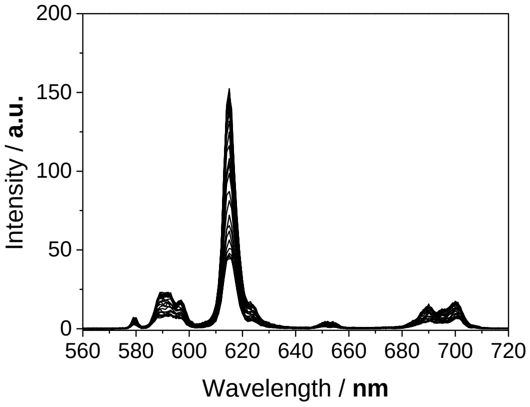

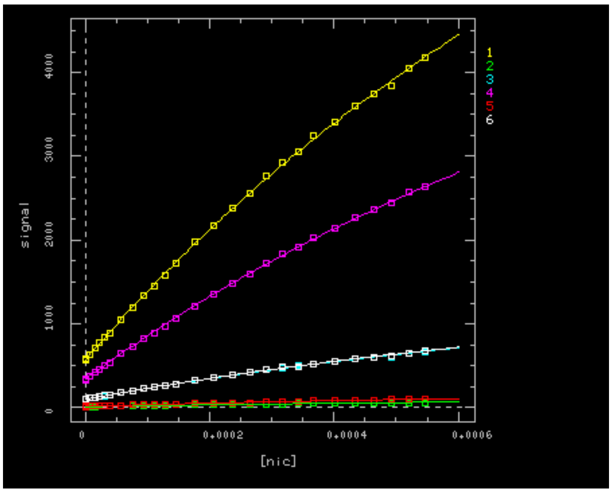

1 mM LiOH in methanol –nicotinate – 25 °C

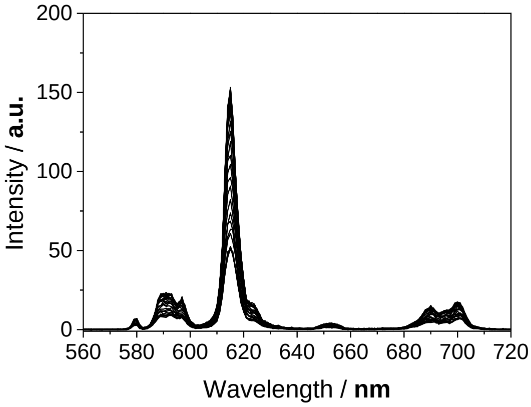

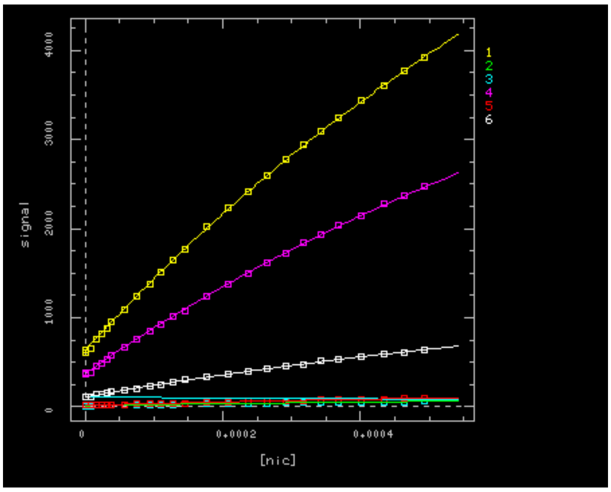

1 mM LiOH in methanol –nicotinate – 30 °C

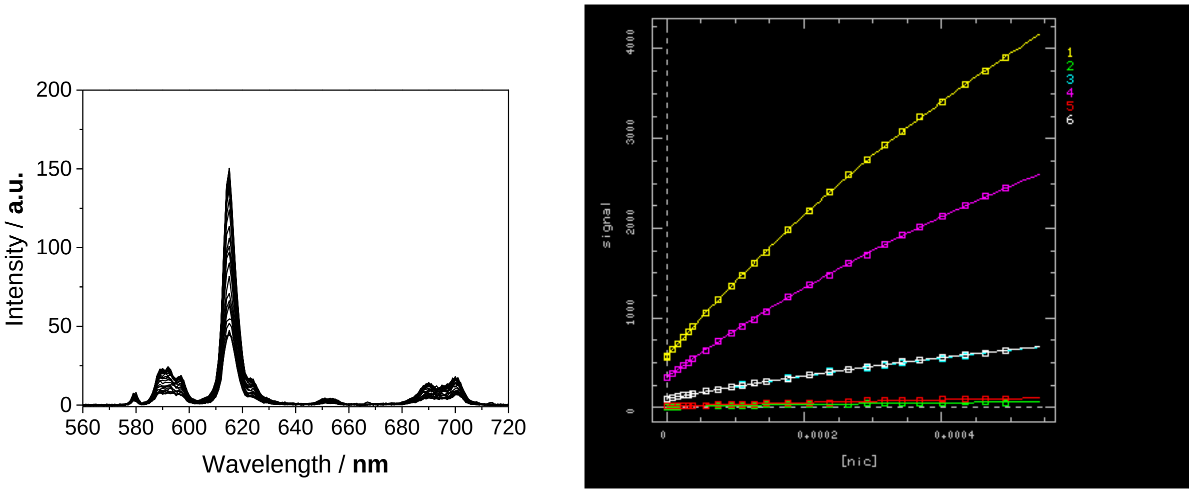

1 mM LiOH in methanol –nicotinate – 40 °C

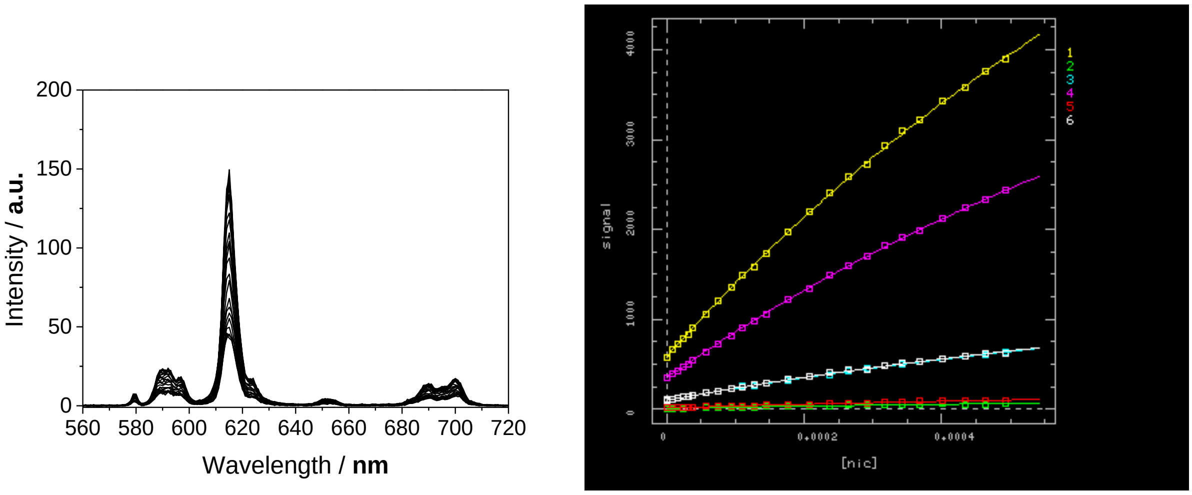

1 mM LiOH in methanol –dinicotinate – 20 °C

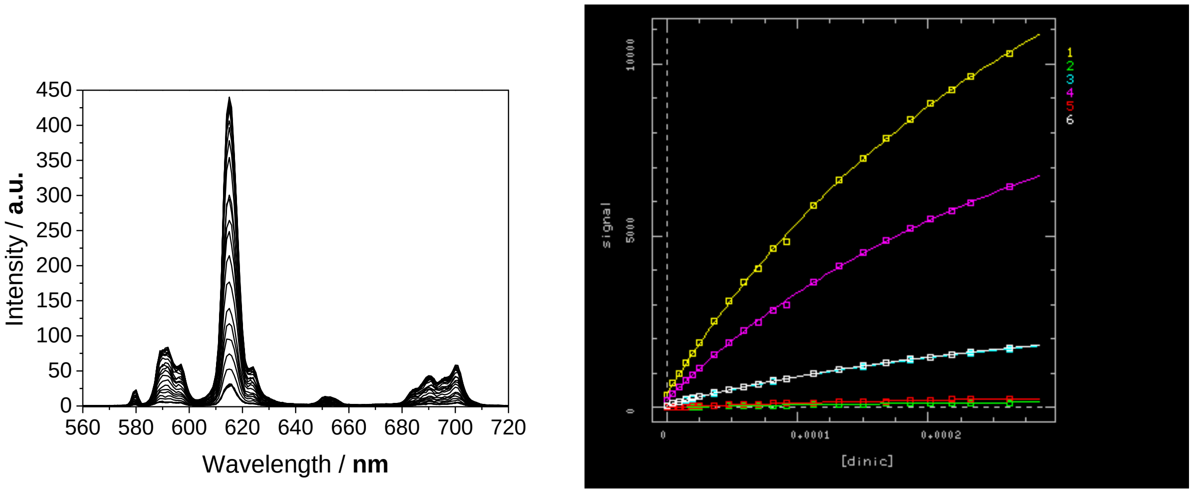

1 mM LiOH in methanol –dinicotinate – 25 °C

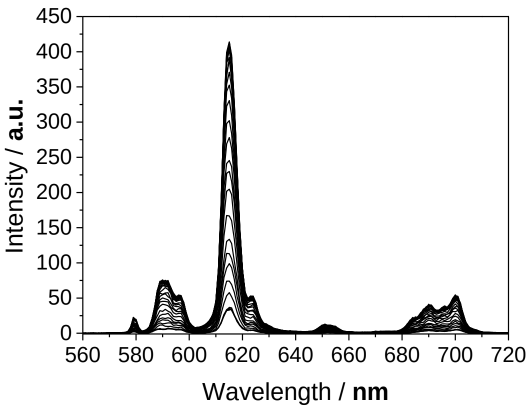

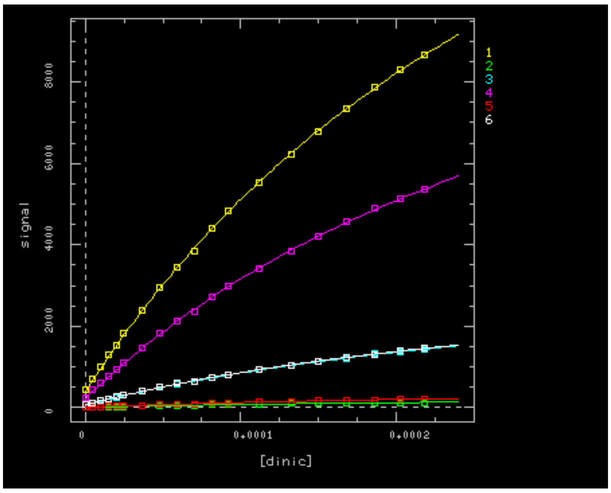

1 mM LiOH in methanol –dinicotinate – 30 °C

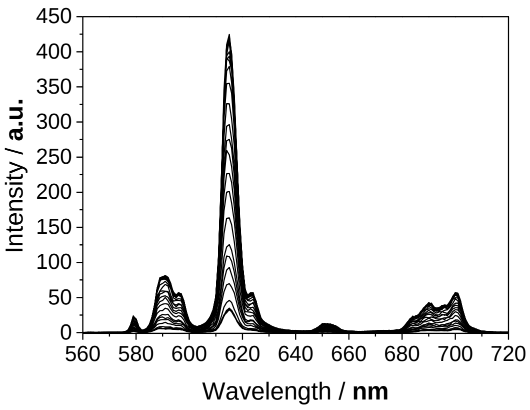

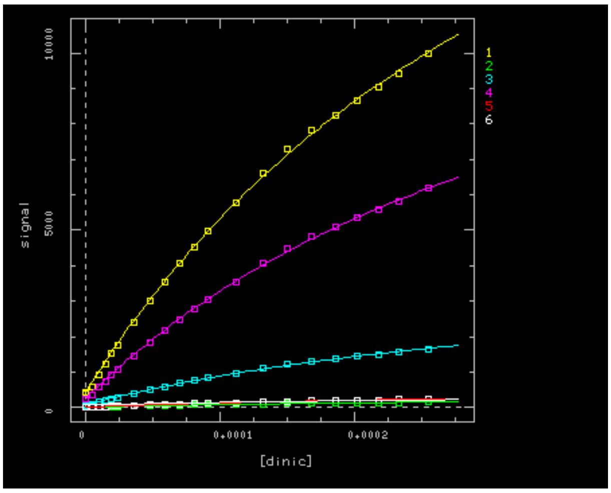

1 mM LiOH in methanol –dinicotinate – 40 °C

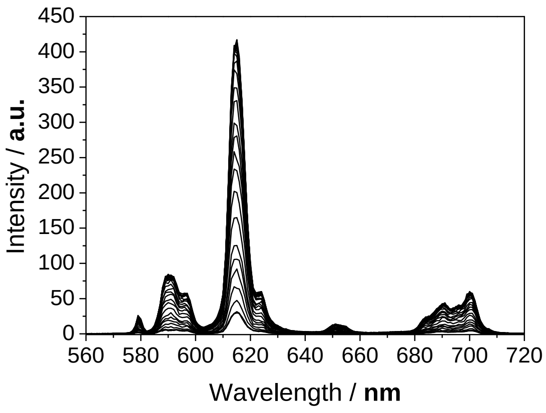

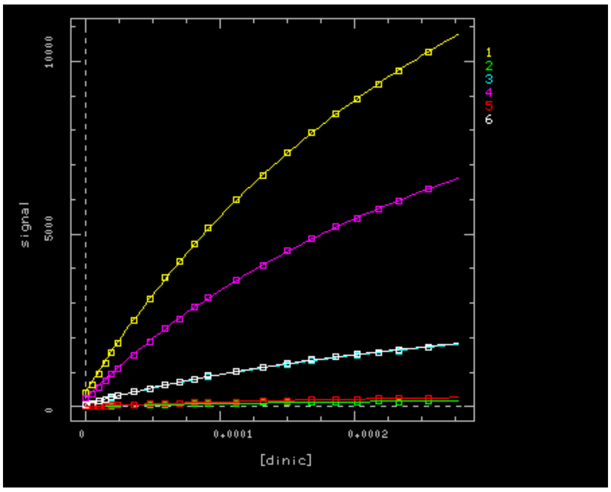

1 mM LiOH in methanol – phthalate – 20 °C

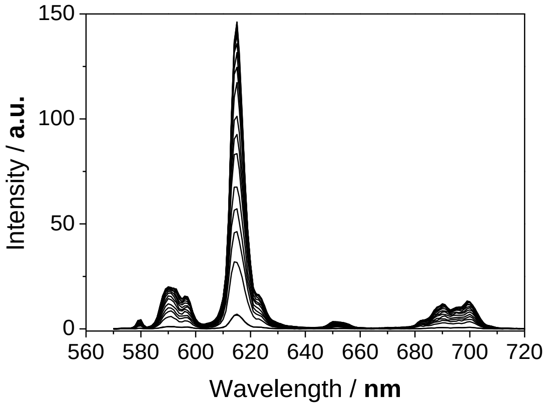

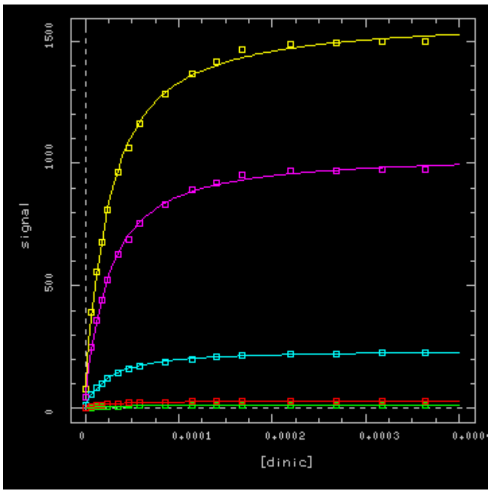

1 mM LiOH in methanol – terephthalate – 20 °C

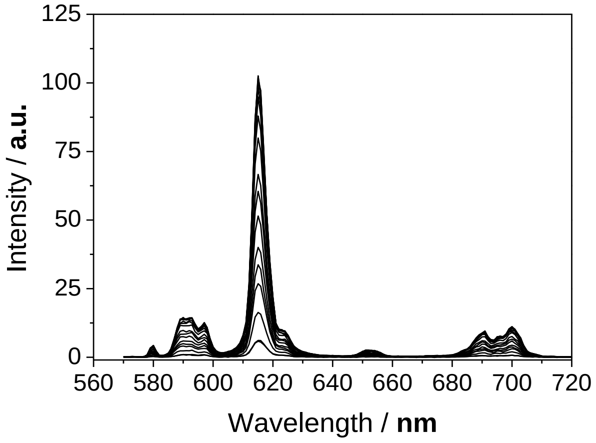

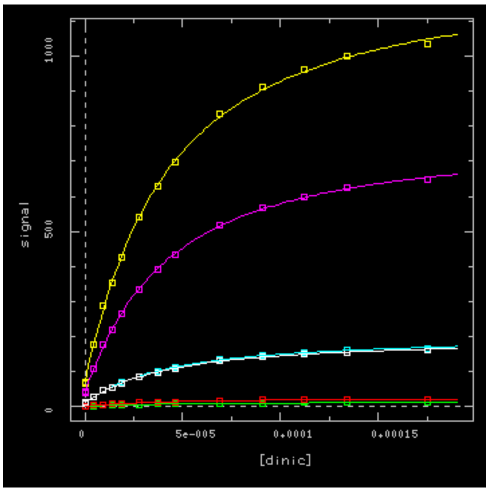

Luminescence decay profiles

Recorded using a Cary Eclipse fluorimeter, with 0.1 ms gate time and 0.2, 0.25 and 0.5 ms decay times, the decays are generated by moving the time window. Decays were recorded following 240 nm excitation with 20 nm excitation slits and 10 nm emission slits. The emission wavelength observed for each decay is indicated on each graph. Temperature control was afforded by a Cary Single-Cell Peltier element.

Pure H_2O and D_2O

All measurements were performed at 25 C. pH and pD of the solution is indicated on the graph.

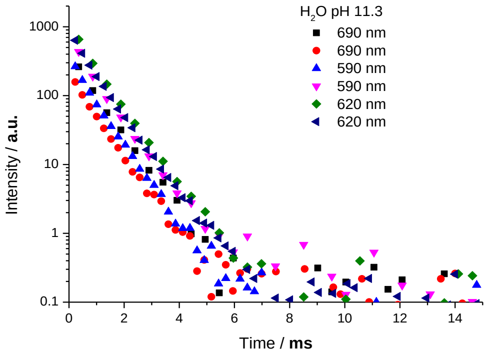

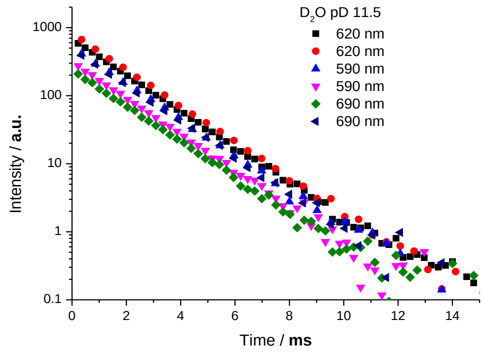

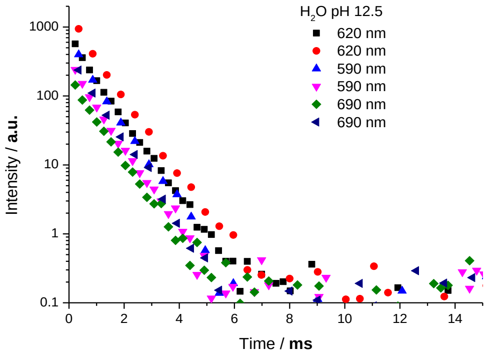

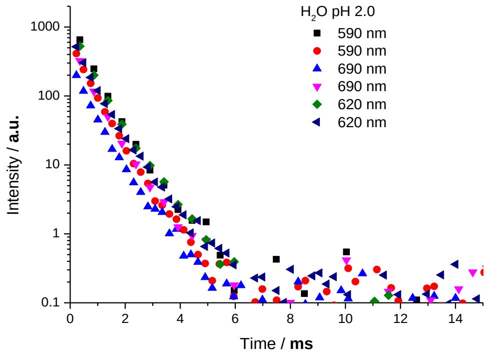

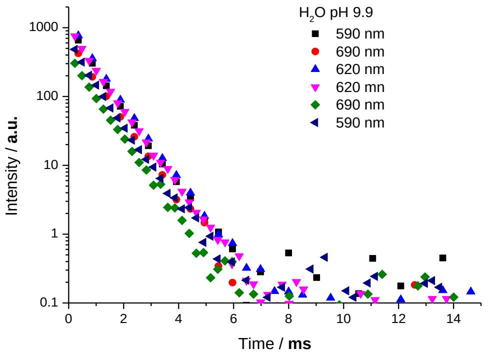

Titrations in buffers

All measurements were performed at 25 C, before and after titrating with dinicotinate. pH of the buffers are: PBS pH 7.4, HEPES pH 7.5, and BBS pH 8.1. Buffer and the presence of dinicotinate is indicated on the graph.

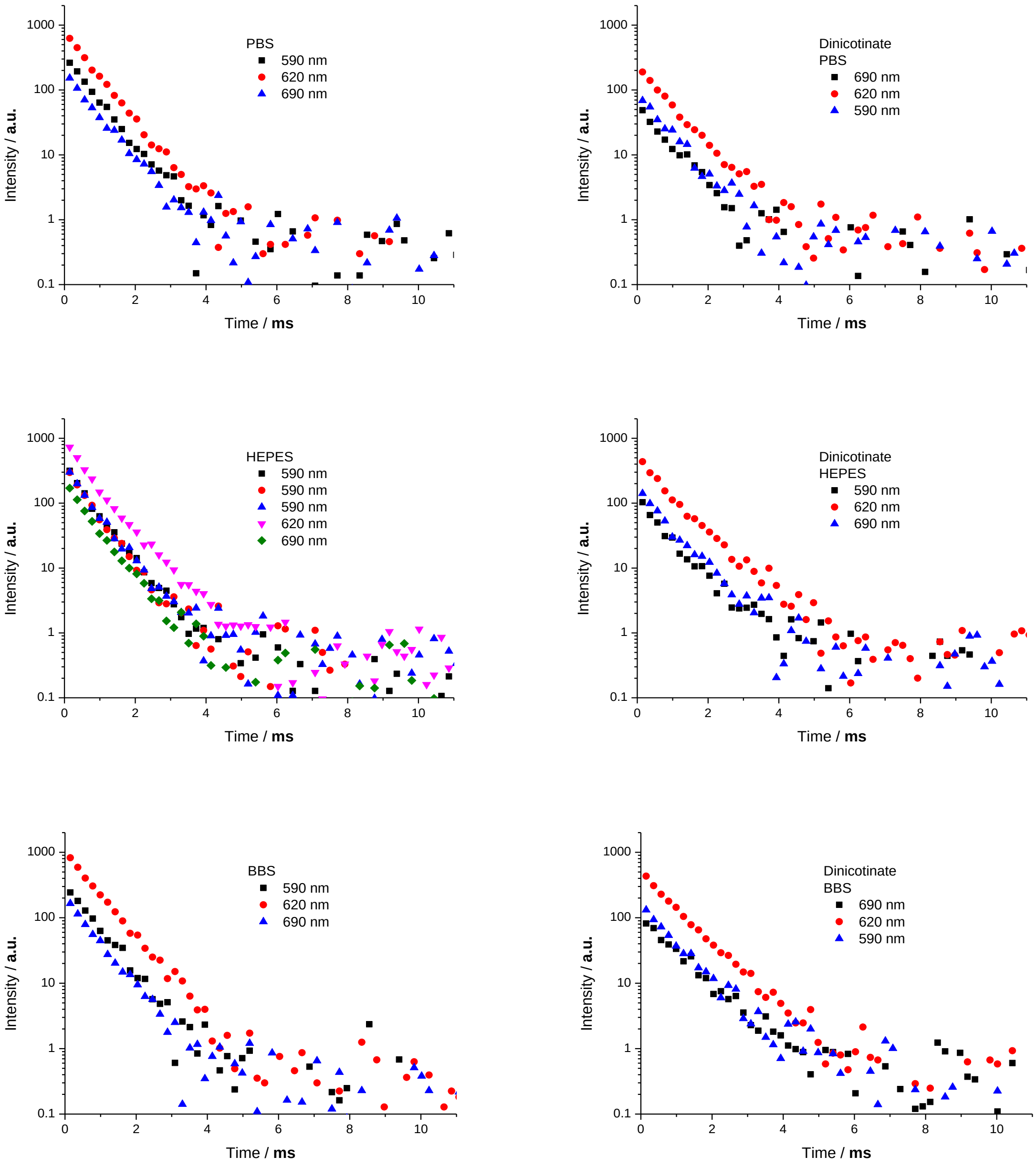
PBS
590 nm
620 nm
690 nm
Dinicotinate
PBS
690 nm
620 nm
590 nm
HEPES
590 nm
590 nm
590 nm
620 nm
690 nm
Dinicotinate
HEPES
590 nm
620 nm
690 nm
BBS
590 nm
620 nm
690 nm
Dinicotinate
BBS
690 nm
620 nm
590 nm
Intensity / a.u.
Time / ms

Methanolic solution

Decay profiles were recorded in methanol with 1 mM LiOH·H_2O, and at the end point of the titration. For isophthalate and methanol the temperature was varied. Temperature and titrant is indicated on the graph.

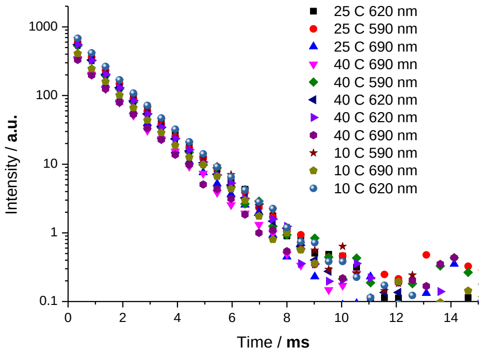

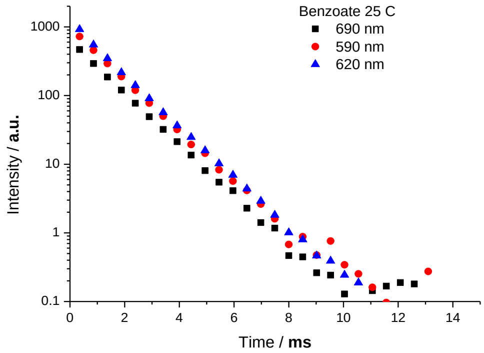

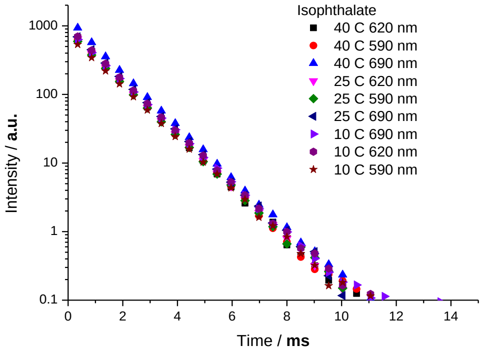

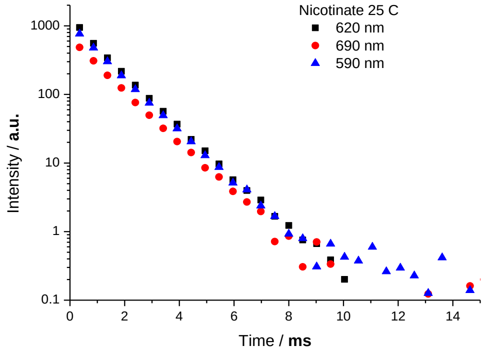

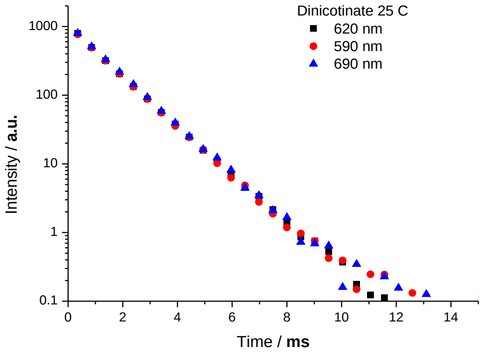

Supplement: Supplementary file 1 — Supplementary [file open0004-0509-sd1.pdf]
